# Roadmap on optical sensors

**DOI:** 10.1088/2040-8986/ad0e85

**Published:** 2023-12-18

**Authors:** Mário F S Ferreira, Gilberto Brambilla, Luc Thévenaz, Xian Feng, Lei Zhang, Misha Sumetsky, Callum Jones, Srikanth Pedireddy, Frank Vollmer, Peter D Dragic, Ori Henderson-Sapir, David J Ottaway, Elodie Strupiechonski, Goretti G Hernandez-Cardoso, Arturo I Hernandez-Serrano, Francisco J González, Enrique Castro Camus, Alexis Méndez, Paola Saccomandi, Qimin Quan, Zhongcong Xie, Björn M Reinhard, Max Diem

**Affiliations:** 1 University of Aveiro, Portugal; 2 University of Southampton, United Kingdom; 3 EPFL, Switzerland; 4 Jiangsu Normal University, People’s Republic of China; 5 Zhejiang University, People’s Republic of China; 6 Aston Institute of Photonic Technologies, Aston University, Birmingham, United Kingdom; 7 Department of Physics and Astronomy, Living Systems Institute, University of Exeter, United Kingdom; 8 University of Illinois at Urbana-Champaign, United States of America; 9 Department of Physics and Institute of Photonics and Advanced Sensing, The University of Adelaide, SA, Australia; 10 OzGrav, University of Adelaide, Adelaide, SA, Australia; 11 Mirage Photonics, Oaklands Park, SA, Australia; 12 CONACYT-Cinvestav-Querétaro, CIDESI, Mexico; 13 Philipps-Universtät Marburg, Germany; 14 University of Warwick, United Kingdom; 15 Ciacyt-UASLP, Mexico; 16 MCH Engineering LLC, United States of America; 17 Department of Mechanical Engineering, Politecnico di Milano, Italy; 18 NanoMosaic Inc., United States of America; 19 Massachusetts General Hospital and Harvard Medical School, United States of America; 20 Department of Chemistry and The Photonics Center, Boston University, United States of America; 21 Northeastern University and CIRECA LLC, United States of America

**Keywords:** optical sensors, optical fibre sensors, THz sensors, specialty optical fibres

## Abstract

Optical sensors and sensing technologies are playing a more and more important role in our modern world. From micro-probes to large devices used in such diverse areas like medical diagnosis, defence, monitoring of industrial and environmental conditions, optics can be used in a variety of ways to achieve compact, low cost, stand-off sensing with extreme sensitivity and selectivity. Actually, the challenges to the design and functioning of an optical sensor for a particular application requires intimate knowledge of the optical, material, and environmental properties that can affect its performance. This roadmap on optical sensors addresses different technologies and application areas. It is constituted by twelve contributions authored by world-leading experts, providing insight into the current state-of-the-art and the challenges their respective fields face. Two articles address the area of optical fibre sensors, encompassing both conventional and specialty optical fibres. Several other articles are dedicated to laser-based sensors, micro- and nano-engineered sensors, whispering-gallery mode and plasmonic sensors. The use of optical sensors in chemical, biological and biomedical areas is discussed in some other papers. Different approaches required to satisfy applications at visible, infrared and THz spectral regions are also discussed.

## 1. Optical fibre sensors

### Gilberto Brambilla^1^ and Luc Thévenaz^2^

^1^University of Southampton, United Kingdom

^2^EPFL, Switzerland

### Status

Optical fibre sensors (OFSs) are devices which exploit optical fibres to monitor physical quantities and provide an output in the electronic domain. In common with other optical sensors, OFSs provide immunity to electromagnetic interference, capability to work in harsh environments and top performance. In addition to other optical sensors, OFSs allow multiplexing to a level which makes them cost competitive with respect to other, non-optical, types of sensors. The global OFS market has continuously grown in the last three decades and was estimated to be in the region of USD 2.7–2.9 B in 2020–2021. It includes a myriad of sensing applications ranging from chemical to physical, from gyroscopes to distributed sensors.

Most of OFSs consist of four components: a transducer, which converts the physical measurand into an optical signal; a detection system, which converts the optical signal into the electronic domain; a waveguide, which delivers light from the source to the transducer and then to the detection system; and a source, which generates the light that will be turned into the optical signal by the transducer. OFSs can be broadly classified in intrinsic and extrinsic sensors by the role that the fibre has in the system: while in the former the fibre itself holds the role of both transducer and waveguide, in the latter it holds solely the role of waveguide. Successful examples of these type of sensors include endoscopes and fiberized optical coherence tomography (OCT). A further classification discerns intrinsic OFSs into distributed, quasi-distributed and point sensors according whether transducing occurs continuously along the whole fibre length, in selected discrete points or in a single point. The most successful distributed sensors include temperature and vibrations, and at a minor extend strain. Fibre Bragg gratings (FBGs) are the most prominent member of quasi-distributed sensors because of their capability to multiplex and measure temperature and strain in multiple locations, especially in the marine environment. Finally, the group of point sensors is very diverse and contains sensors based on various types of interferometers (such as hydrophones and gyroscopes), and sensors relying on the change of the polarization state (current sensors) or of the complex refractive index (bio and chemical sensors).

Overall, the global market for intrinsic OFS is expected to grow strongly over this decade and reach a value of USD 7.2 B by 2030, with an average compound annual growth rate (CAGR) of 11.5% [1]. Although the high-value oil and gas industry currently represent half of the market for intrinsic OFSs and will continue to be a major driving force in the next decade, significant thrust should also rise from homeland security (border control), civil engineering (structural health monitoring), power and utility (power cables monitoring, nuclear fusion), industrial (process monitoring), and defence/aerospace (gyro).

### Distributed sensors

Optical fibres offer the unique property to realise fully distributed sensing, providing a continuous and independent information about an environmental quantity at any position along the fibre [2]. In its most direct implementation, a light pulse is launched into the long sensing fibre and is then continuously back-reflected to the fibre input end through natural scattering processes, like in a radar system. Analysing this back-reflected light (spectrum and amplitude) informs about the quantity to be measured and observing its time response translates into a position-resolved information, considering the finite speed of light and the specific time required by light for returning from a given point along the fibre.

This way such a sensor can substitute for thousands of point sensors, the sensing element having the two functions of converting the measured quantity into a modulation of the signal and of transmitting the signal before and after modulation to the processing unit. It is evident that the optical fibre is an excellent candidate to be such a sensing element, the main difficulty being to identify the right phenomenon activated by the measured quantity that will give the proper modulation on the signal. For this purpose, the three natural scattering processes observed in glass are exploited: historically the first distributed fibre sensor was realised in the late 1980’s using Raman scattering that shows a scattering cross-section significantly dependent on temperature. Such Raman distributed temperature sensors are still implemented to survey the temperature profile of large structures such as hot spots along energy cables and fire detection in tunnels.

A few years later, Brillouin scattering was proposed to realise distributed sensing, showing a spectral signature dependent on temperature and strain. This type of sensor is more sensitive and shows an extended distance range and a better spatial resolution. Hence, it is widely employed in energy industry, infrastructure and environment monitoring, homeland security, etc…

More recently, Rayleigh scattering has been proposed as a more advanced technique, in which the information is obtained by comparing the shape of backscattered traces induced by the random interference of coherent light scattered at inhomogeneity centres inside the fibre core. This results in an interferometric sensitivity that enhances the response by some three orders of magnitude. Such systems are successfully used to sensitively detect vibrations, with applications to intrusion detection and seismic monitoring.

The performance of such sensors is globally measured by the number of resolved points—the distance range divided by the spatial resolution—which is in turn scaled by the signal-to-noise ratio (SNR) of the detected signal. Considering the intrinsic properties of optical fibres, 100 000 points can be resolved in state-of-the-art systems, over a maximum distance range of 100 km limited by natural optical losses. Research efforts manage to improve these figures and to speed up the acquisition time which still takes several seconds.

Multimode and multicore fibres have gained increase attraction for telecom applications and it is reasonable to expect further impact in sensing [3]. Increasing computing power will also facilitate the interpretation of backscattered signal through fast postprocessing of encoded incident signal [4] and further use of artificial intelligence (AI). The range extension will continue, beyond current 170 km [5], allowing for further deployment in geophysics [6], where its range capabilities and high resolution provide a competing edge with respect to other types of sensors.

### Quasi distributed sensors

FBGs represent the vast majority of the quasi-distributed OFS market. FBGs have consistently attracted strong interest because of their long lifetime, high accuracy, compact size, fast response time, and, above all, multiplexing; they have found significant applications in the oil and gas market, in structural health monitoring for aerospace and civil engineering, and in the power industry. The global FBG sensor market size is estimated to be USD 0.4 B in 2022, with a forecasted CAGR of 7.4% up to 2028. This will benefit from the larger market of FBG filters for the telecom market, which is expected to growth at a CAGR greater than 20% over the same period.

There are two major challenges that FBGs will continue to face in the next decade: the relatively high cost of each single grating, which makes the whole system uncompetitive for applications that replace transductors frequently, and the cost of the detection system, significantly higher than that used for competing electronic sensors. Draw tower gratings (DTGs), used in conjunction with OTDR, have emerged as an alternative to FBG wavelength multiplexing, providing a cheaper alternative to FBGs when cheap disposable transducers are required.

Laser inscribed low-loss backscatterers [7] might in the next decade become a competitive technology to DTGs: low-loss backscattering elements are easier to make and cheaper than FBGs or DTGs, and therefore provide a cheaper alternative to FBGs and DTGs. As DTGs, low-loss backscattering elements do not exhibit frequency encoding, thus they need other measurement techniques, such as time of flight—OTDR. The use of low-loss enhanced back-reflection fibres will likely continue to impact also distributed sensing, because of the significant improvement it provides in the signal to noise ratio [8].

The large cost of current detection units is due to the multiple elements present in the sensing system, which relies on the diffraction of light to spatially spread the various frequency components and selectively monitor small wavelength ranges for detection. Cheaper spectrometers have been proposed using speckle patterns [9], and further development into a temporally stable configuration might provide a future solution for an overall cheaper interrogation unit.

Integration will continue to play a significant role, and will be key to decrease cost of both source and detections systems.

### Point sensors

Point sensors include a large variety of sensors, including vibration, temperature, chemical, biological, acceleration and rotation, just to cite a few.

Amongst point sensors, the fibre optic gyroscope (FOG) represents arguably the biggest success, with strong deployment in aerospace and defence. Estimations of the current global FOG market size differs widely, but mostly within the range USD 0.8–1.4 B, with a CAGR of 3.6%–8.1% over the next decade. This is likely to remain the most successful point sensor, because of the increasingly wide deployment in drones, UAVs and of the better gyro performance at smaller rotations, which allow for an increased number of underwater applications. Research for better performance in the angular random walk (ARW) and bias instability regions will continue to involve longer stretches of fibres, better fibre wrapping configurations and more performant lasers. Yet, disruption will likely come from the use of novel fibres, such as multicore fibre or hollow core fibres: nested anti-resonant hollow core fibres (ARHCF) have exhibited an extremely small backscattering, which could be 45 dB smaller than conventional telecom fibres [10], thus allowing for a significant decrease in the ARW.

For the wider group of point sensors, the next decade should see more sensors deployed in spectral regions now considered forbidden because of the current fibres’ poor transmission: chemical sensors will benefit from the extended operation in the mid-IR wavelength region provided by hollow core fibres. The hollow fibre core and the small overlap (often smaller than 10^−4^) of the propagating mode with the optical fibre grass structure allows to use the fibre as a gas chamber with minimal gas volume and optimal overlap between gas and optical mode, thus minimising the amount of gas needed for testing.

### Extrinsic sensors

As in this class of OFs optical fibres only have the task to carry light between source, transducer and detection system, most of extrinsic OFSs are frequently not included in the assessment of the OFS market. Yet, optical fibre endoscopes are often considered an exception, as they represent the first OFS [11], created well before low-loss optical fibres were developed. The global endoscopy devices and equipment market in 2022 is estimated to be USD 7.9 B, with an expected growth to USD 10.6 B by 2026 at a CAGR of 7.7%. The next decade will continue to see significant growth in this market: research in the long wavelength region will increase, as mid-IR cameras with large number of pixels are becoming increasingly cheaper and multicore fibres/fibre bundles with low attenuation at long wavelengths are being developed.

### Concluding remarks

**Figure 1.**
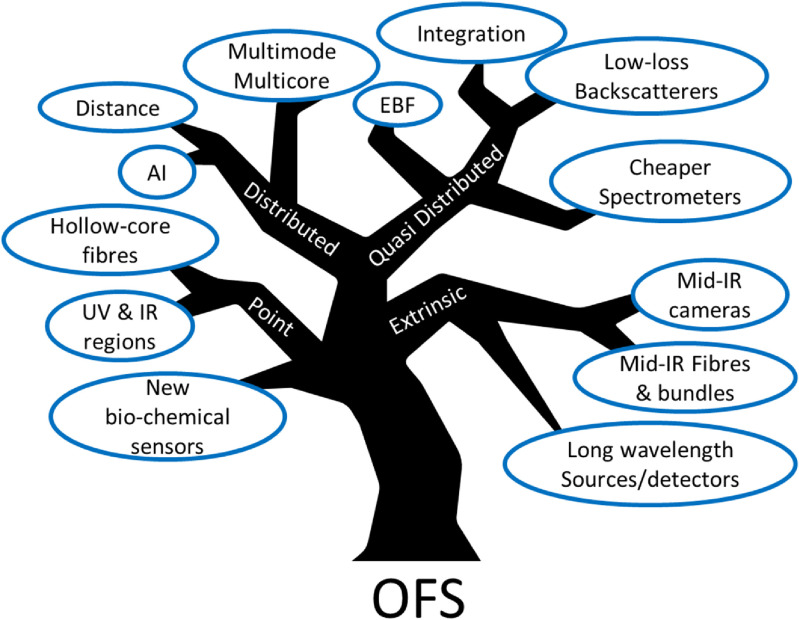
Optical fibre sensor opportunities for development.

Disruptive developments in the OFS field are likely to come from the wide range of directions (figure 1), mostly related to novel fibres. Hollow core fibres, with low attenuation, increased transparency window, small Rayleigh backscattering and minute modal overlap with the fibre glass structure will promote new developments in gyroscopes, sensors for harsh environments and nuclear fusion. Multicore fibres will provided additional referencing and might result in advantageous performance in shape sensing and gyros. Enhanced backscattering fibres will extend the sensing range beyond 200 km from a single end, increasing the deployment in marine environments and earth science.

The thrust for cheaper optical sources and detectors will continuously decrease the cost of OFSs, making them competitive for a wider range of applications now dominated by other forms of sensors. Cost competitiveness could open a new field of fibre sensors to the home, where the broad deployment of optical fibres in residential settings might allow for their prompt use in sensing.

### Acknowledgments

The authors acknowledge funding from EPSRC (Grant EP/S013776/1), The Royal Society (London) (CHL\R1\180350), and NERC (NE/S012877/1).

## 2. Specialty fibres for sensing applications

### Xian Feng

Jiangsu Normal University, People’s Republic of China

### Status

Historically, specialty fibres have experienced a spiral rising evolution, from using simple core/cladding structure and simple-material [12] (e.g. silica and other non-silica glasses, which were well developed before the burst of the telecom bubble), to introducing wavelength-scale microstructure features [13], and at the latest integrating multi-material, multi-structure, and multi-functionality in the fiberized platform [14].

An optical fibre sensor is for detecting the physical or chemical information in the surrounding environment. The fibre output signals contain the information in either spatial, temporal, frequency, polarization, or phase domain, due to the interactions between the propagating optical modes along the fibre core and the external fields. Three fundamental components, the light source, the fibre medium (i.e. sensing element), and the detector (i.e. translator), are necessary for fulfilling the desired sensing function. Specialty fibres are advantageous over the conventional optical fibres, due to their tailored fibre material(s) and structures for the enhancement of the interaction between the optical modes within the fibre and the external fields [14].

**Figure 2.**
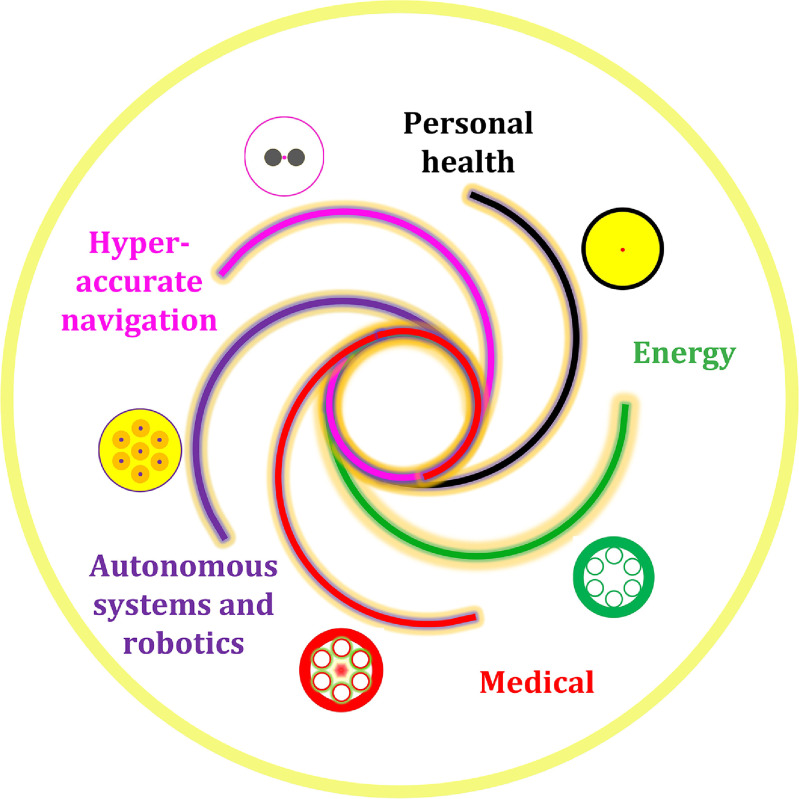
Selected applications of specialty fibre sensing for critical and emerging technologies.

Due to the compactness and the flexibility, specialty fibres are widely used for many advanced sensing areas (see the schematic plot of figure 2), including personal health, energy, environment, the emerging pathogens detection and characterization, autonomous systems and robotics, and hyper-accurate positioning, navigation, and timing (PNT). Most of those areas can be assigned into the category of critical and emerging Technologies.

### Current and future challenges

The challenges to specialty fibre sensors are mainly from the practical demands, while there are many other counterpart solutions. One of the most competitive technologies is the electronic chip sensors, originating from the semiconductor industry and are capable of directly generating electrical signals by the external stimuli. The competition with these counterpart technologies and the perform-or-perish trend applies high pressure on the development of specialty fibre sensing technology.

A comprehensive coverage of the current and future challenges for specialty fibre sensors will not be possible due to the ignorance and limited expertise of the author. Nevertheless, this section tries to address this issue by highlighting some recent hot subjects in the following:

(i) Wearable products have been widely commercially available, for the usage of monitoring personal daily activities and health. With the assistance of many embedded sensors, such products are capable of monitoring heart rhythms, blood oxygen saturation, blood pressure, and so on. Since these components have limited contact area with human’s surface area, only limited physical information of human body can be retrieved.
(ii) In terms of the energy applications, lithium batteries, which have high charge density and disposability, are widely used in smartphones and electric cars. However, fire and explosion could occur when the Li batteries start degrading after being used for a certain period.
(iii) As the largest pathogen disease in this century, SARS-CoV-2 Coronavirus (COVID-19) disease has caused confirmed cases over 0.5 billion and deaths over 6 million. Due to the rapid spread rate of the disease and the large number of the infections and deaths, a fast, accurate and economic testing approach are crucial for the mass surveillance of SARS-CoV-2 infections. The traditional nucleic acid test still need long waiting time for results.
(iv) The cutting-edge robotics and autonomous systems require sensors for real-time position, shape, posture tracking.
(v) PNT is commonly provided by global positioning system (GPS) constellations. However, GPS becomes problematic, for example when the user is inside an underground tunnel or in a submarine or even for rapidly developing autonomous underwater vehicles. The rising geopolitical conflicts lead into the real concern that such satellite-based systems could be severely interferenced or damaged, making the systems useless. A self-sufficient navigation system that can aid navigation for both civilians and military users, in case that GPS is catastrophically crashed.

### Advances in science and technology to meet challenges

(i) Distributed smart clothes made of multiplexed optical fibres have been developed as a multimodal wearable sensor for on-site detecting multiple physical or chemical parameters of large area of human body [15].
(ii) Either hollow-core fibre sensors or evanescent-field tapered fibre sensors can be embedded into the inner Li-battery for spectral in-situ analysis. Hollow-core optical fibre sensors have been demonstrated for operando Raman spectroscopic investigation of Li-ion battery liquid electrolytes. The hollow-core fibre functionalizes not only as the microfluidic channel to sample electrolyte liquid with a volume of *μ*l but also as the waveguide to send the excitation laser signal in and retrieve the Raman signal out [16].
(iii) The specialty fibre based virus sensors require the assistance of a certain enhancement mechanism because of the sub-nanometre size of viruses and the weak signals. A fibre-based surface plasmon resonance (SPR) sensor can enhance the weak signals generated from the interaction between the excitation laser and the viruses by a few orders of magnitude. For such fibre sensors, D-shaped fibres or hollow-core fibres are normally used. Nanostructured metal features are deposited, either on the outer surface of the D-shaped fibre or on the inner core surface of the hollow core fibre. A relatively fast response time of ∼10 min can be obtained to verify a positive virus carrier, in comparison with the typical 3–4 h of performance time when the traditional method is used [17].
(iv) FBG arrays inscribed in multicore optical fibres have been proven to be a powerful tool for sensing 2D and 3D curvature and shape with high resolution and accuracy. Hence it enables the cutting-edge applications for example smart robotic surgery [18, 19].
(v) The geomagnetic navigation technology is a promising alternative for GPS navigation, because the earth’s magnetic field is the inherent feature of the earth and can be mapped and tracked [20]. The existing magnetic sensors have many shortcomings, including low sensitivity, large volume, high power consumption, which do not fit the requirements of long-term underwater operations. One of the effective technical solutions should be fibre magnetic-field sensors utilizing magneto-refractive properties of rare-earth doped glasses [21]. With a proper selection of paramagnetic rare-earth dopants and optimized fabrication controlling for achieving low-loss fibre, highly sensitive magnetic field detection with sensitivity of fT-level should be realized using hundred-meter-long highly birefringent fibre.

### Concluding remarks

The rapid change of the modern society provides great challenges but also great opportunities for the development of specialty fibre sensors. The ultimate strategy of future specialty fibre sensor technology to deal with the challenges should be to balance the combination of materials and structures for achieving the desired sensing functionalities [14].

### Acknowledgments

This work is supported by the National Natural Science Foundation of China (NSFC, 62175096), Jiangsu innovation and entrepreneurship Team, Priority Academic Program Development of Jiangsu Higher Education Institutions, and Jiangsu Collaborative Innovation Centre of Advanced Laser Technology and Emerging Industry.

## 3. Micro- and nano-engineered sensors

### Lei Zhang

Zhejiang University, People’s Republic of China

### Status

Back in 1966, Kao and Hockham initiated low-loss optical fibres, which had quickly found extensive applications in optical communication and sensing. To date, distributed fibre-optic sensors, photonic crystal fibre sensors, and chalcogenide glass fibre sensors have been extensively studied and found various applications. With the rapid progresses in nanotechnology and flexible opto-electronics, there is an increasing demand for high performance sensors with faster response, smaller footprint, higher sensitivity, and lower power consumption to explore the limit of detection of force or the interactions between molecules to understand the fundamentals of physics, biology, and medical science, which spurred great efforts for micro and nano-engineered optical sensors. Since the probing light wavelength is close to or below the dimension of the micro and nano-engineered structures, these sensors offer more flexibility in tailing light for sensing weaker light–matter interactions.

Since the first demonstration of subwavelength-diameter silica micro/nanofibers (MNFs) for low-loss optical waveguiding in 2003, MNFs have attracted considerable attention due to engineerable strong evanescent fields and excellent mechanical properties, which makes them ideal building blocks for waveguiding sensors on micro/nano scale. To assemble an MNF based sensor, an MNF should be well packaged to avoid environmental disturbance or surface contamination. Benefiting from the networks of microchannel, the optofluidic system can protect the MNFs from unintended stimulation, provide small volume of sample for the MNF, and renew the MNF surface, making the MNF suitable for detect ultratrace molecules in solution [22]. On the other hand, the embedded MNF is a multifunctional detector for real time monitoring the microflow status, which is important for the feedback control of an optofluidic system [23].

In addition to the waveguiding structures, the resonant structures can significantly enhance light-matter interactions, making them ideal candidates for highly sensitive sensors. For example, optical whispering-gallery mode (WGM) microresonators (e.g. microspheres, microdisks, and microtoroids), confining resonant photons in a microscale volume, have been used for the detection of materials in different phases and forms, including gases, liquids, and chemicals [24]. Different from the WGM resonators, metal nanostructures (e.g. noble metal nanoparticles) provide a mode size much smaller than the vacuum wavelength of the light and comparable with the cross section of biomolecules, making them favourable for single molecule or particle sensing. Overall, both waveguiding and resonating sensors hold great potential for next generation sensing applications.

### Current and future challenges

In the past few years, we have witnessed the success in micro- and nanoengineered optical sensors; however, more challenges may come from fabrication, practical applications, and sensing mechanism innovations. Firstly, from the fabrication side, as the feature sizes go down to subwavelength scale, the high precision, cost effective and scalable fabrication technique is a key issue related to the sensing performance and potential for practical applications. For example, how to draw MNFs with controlled diameter, functionalize MNFs with high repeatability, and automatically package MNFs with high robustness remain challenging. For the high quality WGM resonators, delicate fabrication process, expensive instruments, and complicated coupling process limit their applications in both scientific research and practical applications. Noble-metal nanocrystals represent an important class of materials for localized SPR (LSPR) and surface-enhanced Raman spectroscopy (SERS) based sensing. To move from academic studies to practical applications, one has to address the issue of scaling up a small batch-based synthesis of the nanocrystals.

Secondly, from the application side, there are two typical areas: scientific research and practical applications. For example, when the detection limit of microforce is down to fN level or smaller, the sensor can be used to measure of critical Casimir forces, optical scattering forces, and optical momentum. With the rapid development of in health care, energy, robotics and AI, there is an increasing demand for novel sensors to meet the need from these areas. For example, electronic skin (E-skin) can simultaneously differentiate among various physical stimuli from the complex external environment, however, its ultimate performance is fundamentally limited by the nature of low-frequency AC currents.

Thirdly, to meet the challenges in the abovementioned cutting-edge applications, new sensing structures and sensing mechanisms are highly desired. For example, current leakage due to insufficient insulation, and high sensitivity to electromagnetic disturbances are still challenges for E-skin sensors. An alternative to E-skin is the detection of pressure, strain, bending, and temperature by optical sensors due to their inherent electrical safety, immunity to electromagnetic interference, and small size. Note that multiparameter (e.g. pressure, strain, and temperature) signals often mix together, how to realize an efficient decoupling of the output of a fibre-optic sensor should be considered for real applications.

### Advances in science and technology to meet challenges

From the fabrication side, to control MNF diameter, the cutoffs of high-order modes were real time monitored during the fibre-pulling process. By accurately measuring the time interval between two drops, the diameter precision can be less than 2 nm with a transmission as high as 99.4% [25]. To address the challenge faced by the inorganic microcavities, polymers, such as poly(methyl methacrylate) (PMMA), epoxy resin, and SU-8, have received considerable attention due to their potential for devices with advanced functionalities not attainable by inorganic materials [26]. To scale up the production of noble metal nanocrystals, continuous flow synthesis based on droplets has proved to be an effective platform for large scale synthesis of shape-controlled nanocrystals.

**Figure 3.**
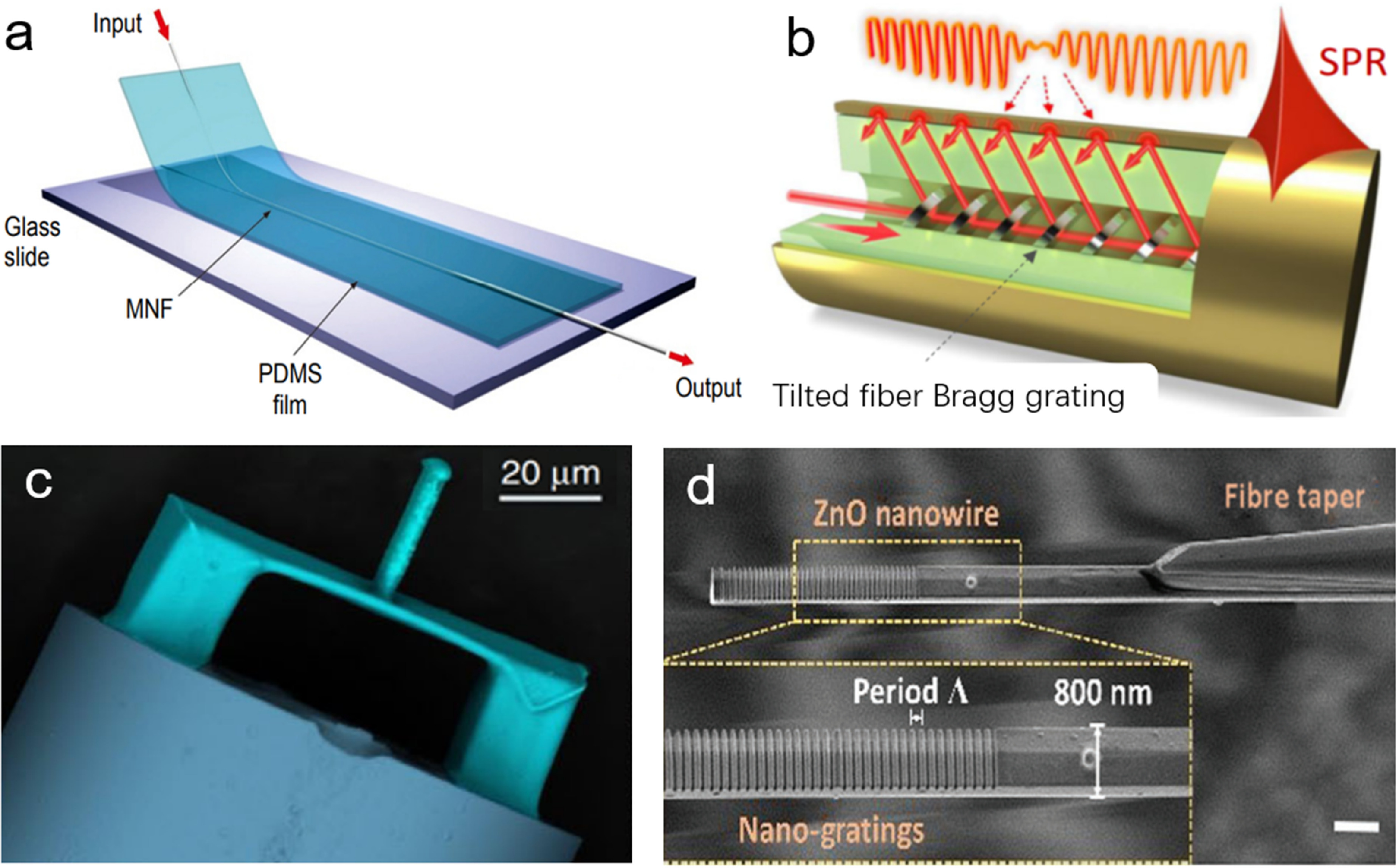
Typical mico- and and nanostructures optical sensors. (a) Schematic of an MNF enabled optical skin. Reproduced from [27]. CC BY 4.0. (b) Schematic of a tilted fibre Bragg grating sensor. Reproduced from [28]. CC BY 4.0. (c) SEM images of fabricated polymer clamped-beam probe on the fibre tip. Reproduced from [29]. CC BY 4.0. (d) SEM image of the fibre sensor, with a nanowire diameter of 800 nm. Scale bar, 1 *µ*m. Reproduced from [30]. CC BY 4.0.

To meet the increasing demand for novel applications, there have been a great number optical fibre sensors have been reported recently. For example, to overcome the limitation of face by E-skins, MNF was used to assemble ultrasensitive optical skin sensors (figure 3(a)), which can detect weak pressure with ultrahigh sensitivity (1870 kPa^−1^), low detection limit (7 mPa) and fast response (10 *μ*s) [27]. To understand ion transport kinetics and electrolyte-electrode interactions at electrode surfaces of batteries in operation, an optical fibre plasmonic sensor capable of being inserted near the electrode surface of a working battery was demonstrated (figure 3(b)) [28].

Furthermore, new sensing mechanisms or structures could be introduced into optical sensors with micro- or nanoengineered structures. For example, optical fibre tip devices have miniature sizes, diverse integrated functions, and low insertion losses, making them suitable for high-sensitivity nanoforce measurements (figure 3(c)) [29], in situ early monitoring of cellular apoptosis (figure 3(d)) [30], cancer sensing and therapy [31]. Although tremendous efforts have been made in developing novel nanomaterials/nanostructures for high performance sensors and environmental remediation [32, 33], nanosafety is paramount considering the risks associated with manufactured nanomaterials [34, 35].

### Concluding remarks

The development of micro- and nanoengineered structures enable optical sensors with improved performance, in terms of footprint, sensitivity, and response time, that are not possible with conventional optical sensors. Some important progress has been made and further new advances are expected in the areas of ultrasensitive optical force sensors, wearable sensors, and optofluidic-chip-based sensors. The practical realization of micro- and nanoengineered sensors requires advances in the fabrication and integration techniques, a better understanding of multidisciplinary sciences, and taking advantage of new physical effects.

### Acknowledgments

This research was supported by National Natural Science Foundation of China (No. 61975173), Major Scientific Research Project of Zhejiang Lab (No. 2019MC0AD01), and Key Research and Development Project of Zhejiang Province (No. 2021C05003).

## 4. Whispering gallery mode sensors: towards spatially resolved and spatially independent detection

### Misha Sumetsky

Aston Institute of Photonic Technologies, Aston University, Birmingham, United Kingdom

### Status

**Figure 4.**
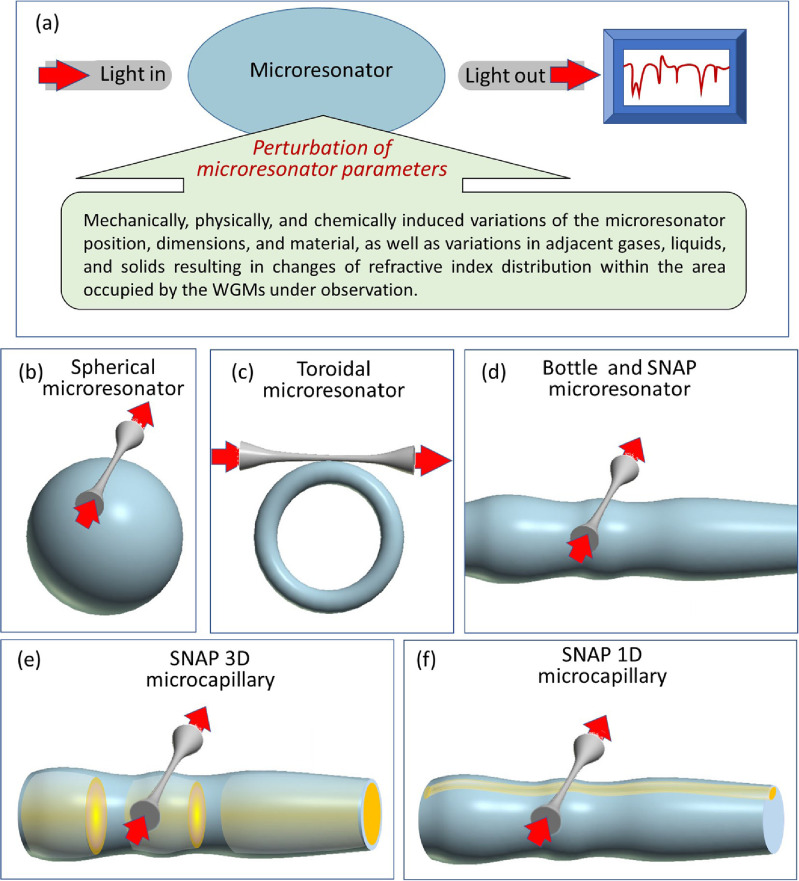
(a) Illustration of sensing with a WGM microresonator. Light is evanescently coupled into the microresonator from an input waveguide and collected by the same or different output waveguide. The resonant spectrum of light is measured by the optical spectrum analyser. (b)–(d) Spherical, toroidal and bottle (SNAP) microresonators. (e) 3D SNAP microcapillary with a liquid droplet inside. (f) 1D SNAP microcapillary with liquid inside.

The emerging field of optical microresonators includes research and development of individual and coupled planar and essentially three-dimensional microresonators devices. The general functional scheme of a microresonator device is shown in figure 4(a). The performance of these devices is usually characterised by the spectrum of output resonant light. In contrast to optical signal processing and spectroscopic applications, for sensing applications, the microresonators are designed (e.g. specially shaped and coated) so that their optical parameters are sensitive to variations of selected material characteristics within their volume and closely adjacent medium [36]. Due to the large Q-factor of a broad range of optical microresonators, their resonant spectra can be very sensitive to these variations.

Whispering gallery mode (WGM) microresonators having the characteristic shapes of a sphere (figure 4(b)), toroid (figure 4(c)), and bottle (figure 4(d)) represent a key class of microresonator sensors [36]. Their importance is caused by a relatively large surface area open to the environment, which can be probed by evanescent WGM tails, and exceptionally large Q-factors. For practical applications, these microresonators can be placed on a chip and coupled to robust input-output optical waveguides rather than microfiber tapers shown in figure 4 [37]. The WGM optical sensors can be divided into those detecting changes at the microresonator surface (e.g. appearance and variation of properties of micro/nanoparticles (NPs) with dimensions down to a single atoms and molecules) and changes of the bulk microresonator material parameters (e.g. temperature and stress). Of special importance are the elongated and shallow bottle microresonators, also called SNAP microresonators (SMR), shown in figure 4(d) [38]. A particular advantage of SMR is that they can detect changes at their external surface as well as (similar to bubble microresonators [36, 38]) at the internal surface if fabricated of thin wall microcapillaries (figures 4(e) and (f)). In the latter case, these devices act as microfluidic sensors.

The goal of this roadmap is to discuss several situations when application of WGM microresonators can be beneficial compared to other sensing methods with the examples based on applications of SMRs.

### Current and future challenges

Most of microresonator sensing methods developed to date detect environmental changes with the spatial precision that does not go beyond the characteristic microresonator dimensions. For example, it is commonly suggested that a resonance shift caused by a microparticle indicates on its appearance or displacement rather than its actual coordinates at the microresonator surface [36]. However, in certain cases, more detailed spectral analysis allows us to obtain the information about the microparticle location. For example, the absence of shift of one of the spectral resonances as opposed to finite shifts of others may suggest that the microparticle is situated at the node of the corresponding eigenstate.

Is it possible to develop a comprehensive approach that can give us the detailed information about the spatial distribution of changes happening within the microresonator volume and adjacent medium? Generally, the information contained in the resonant spectra measured as a function of time at the fixed input–output waveguide position, or even continuously as a function of waveguide coordinate and time, is not sufficient to restore the spatial distribution of the microresonator refractive index and its shape. Nevertheless, looking for the situations when this problem can be solved is of great interest.

Alternatively, we may be interested in detecting physical and chemical processes happening with micro/NPs and molecules rather than their positions. In this case, variation of the resonant spectrum caused by these processes, which takes place at the background of the displacement of individual particles, should be extracted. Solution of this problem is more challenging than just detecting the microparticle positions noted above. Nevertheless, we show below that this problem can be addressed easier for specially designed shapes of SMRs.

### Advances in science and technology to meet challenges

Here we present potentially feasible approaches to address the problems of spatially resolved and spatially independent sensing based on the recent progress in the development of the SMR theory [39, 40] and new methods of SMR fabrication [41–43].

We start with the simplest problem of sensing the evolution of a heated droplet in a silica microcapillary illustrated in figure 4(e). It was shown in [41] that a water droplet induces a SMR inside a silica microcapillary which axial width is equal to the width of the droplet. In particular, the reduction of the droplet width due to the evaporation was detected with nanometer accuracy. It follows from the results of [41] that the displacement of the droplet edges along the microcapillary can be restored with the same nanometer precision by monitoring the WGM transmission spectrum for a fix position of the input-output waveguide.

**Figure 5.**
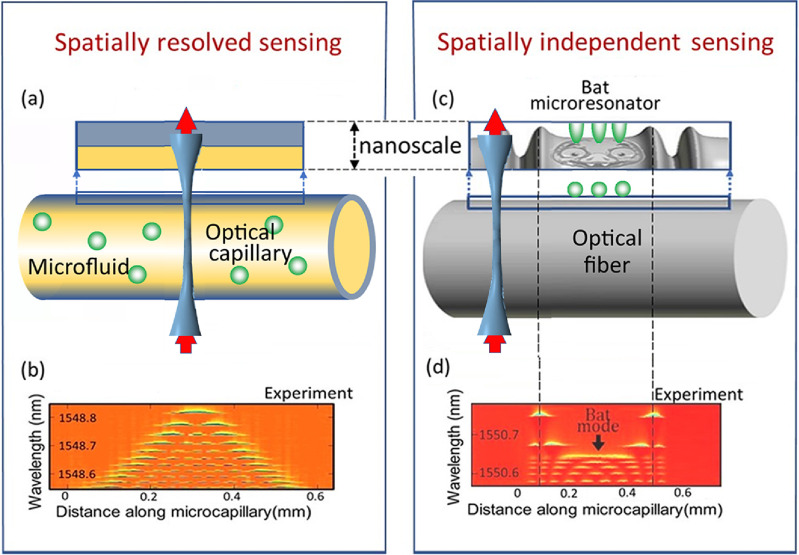
(a) Spatially resolved sensing and manipulation of microfluid components inside a microcapillary. Sensing employs an SMR introduced along the extended length of the microcapillary. (b) The measured spectrogram of such microresonator fabricated in [42]. Reproduced from [42]. CC BY 4.0. (c) Illustration of a bat microresonator. (d) The measured spectrogram of a bat microresonator fabricated in [43]. © [2021] IEEE. Reprinted, with permission, from [43].

A more general approach to detect the microfluidic components in an optical microcapillary suggested in [39] is illustrated in figure 5(a). In this case, sensing of micro/NPs floating in liquid can be accomplished with a specially designed SMR. The spectrogram of such SMR recently fabricated in [42], which is extracted from figure 5(d2) of the latter paper, is shown in figure 5(b). When a microparticle enters and moves inside the volume of SMR close to its internal surface (the latter proximity can be ensured by employing a 1D microfluidic channel illustrated in figure 4(f)), we sequentially observe shifts of eigenwavelengths of wider eigenstates followed by shifts of eigenwavelengths of narrower eigenstates. Observation of the WGM transmission spectrum of the SMR with an input-output microfiber positioned in its centre potentially allows us to determine the axial displacements of a single and several micro/NPs. The proof-of-concept experimental demonstration of such nonlocal microfluidic sensing has been recently presented in [44]. Similarly, the knowledge of the WGM transmission spectrum allows us to determine the SMR profile, spatial temperature distribution (rather than the average temperature [36]) and distribution of irreversible temperature-induced material changes [42] by solving the inverse problem [39]. It was suggested in [39] that WGMs appropriately populated with light can be used as microscopic optical tweezers manipulating microparticles.

Alternatively, a feasible solution to separate the effect of microparticle displacement on the observed SBM spectrum from more complex physical and chemical processes happening with a single or several microparticles was recently proposed in [40] where SMRs having an eigenstate with uniform field amplitude along extended part of the SMR surface area were designed. These SMRs, illustrated in figure 5(c), were called the bat microresonators since the profile of its original design [40] resembled the profile of a bat. A bat microresonator with the spectrogram shown in figure 5(d) was experimentally demonstrated in [43]. Displacement of a microparticle along the surface area with a uniform eigenstate amplitude can be separated since it does not perturb the corresponding eigenwavelength (in contrast to other eigenwavelengths of this SMR) unless the microparticle changes its structure or orientation. We suggest that the bat SMRs can find important applications in sensing as well as in quantum technologies where positioning of maximum possible number of quantum emitters at the resonantly enhanced and spatially uniform region of light is required [45].

### Concluding remarks

It is challenging and often impossible to reveal the details of processes taking place at the optical microresonator from its transmission spectra. However, in special cases, characteristics of sensing objects can be extracted from the resonant spectra with exceptional precision. In this roadmap, we discussed prospects of sensing microfluidic components, such as micro/NPs, and the feasibility of independent detection of their displacement, as well as detecting the spatial distribution of temporal and irreversibly induced material changes along the specially designed SMRs.

### Acknowledgments

The author acknowledges support from the Engineering and Physical Sciences Research Council (Grants EP/P006183/1 and EP/W002868/1), Wolfson Foundation (Grant 22069), and Leverhulme Trust (Grant RPG-2022-014).

## 5. Single-molecule whispering-gallery mode sensing at quantum limits for investigating photocatalytic reactions and key processes in quantum biology

### Callum Jones, Srikanth Pedireddy and Frank Vollmer

Department of Physics and Astronomy, Living Systems Institute, University of Exeter, United Kingdom

### Status

**Figure 6.**
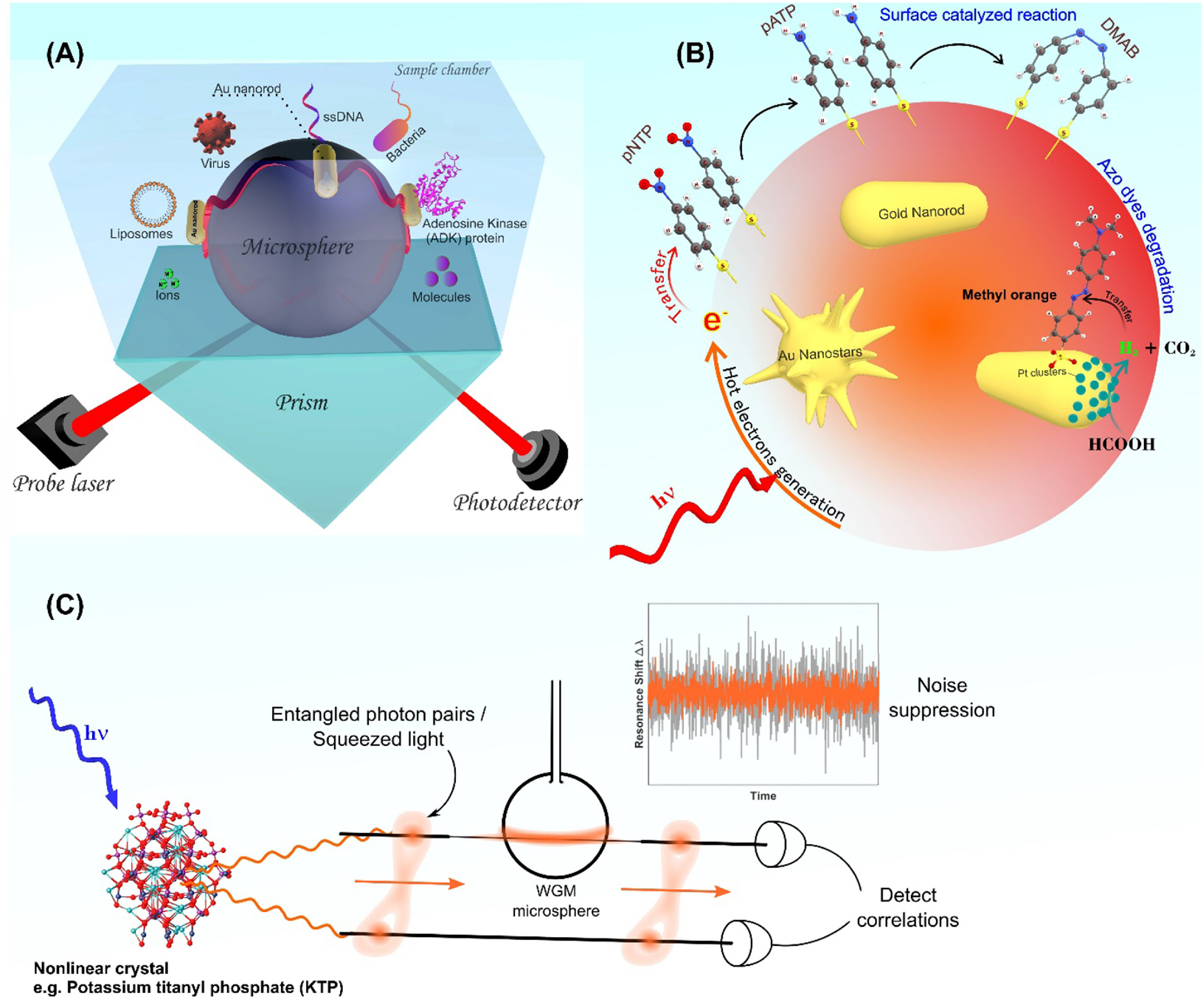
Experimental setup of the plasmon-enhanced optoplasmonic WGM platform. The optoplasmonic WGM sensor is an approx. 100 um diameter glass microsphere with attached gold nanorods. (A) A prism coupler is used to excite WGMs in the glass microsphere. The sample chamber is made in Polydimethylsiloxane (PDMS) and filled with aqueous buffers to detect analytes ranging from bacteria and viruses to activity of the enzyme such as adenosine kinase (ADK). (B) Schematics showing the hot-electron mediated surface catalytic conversion of p-nitrothiophenol (pNTP) to p,p′-dimercaptoazobenzene (DMAB) via p-aminothiophenol (pATP) molecules. (C) Schematic of quantum enhanced sensing using a WGM microsphere. Entangled photon pairs or squeezed light generated in a nonlinear interaction (e.g. using a nonlinear crystal such as KTP) would make possible many different measurement schemes. In all cases, the aim is to suppress the noise level in a WGM measurement, per photon used.

WGM Sensors (figure 6(A)) utilise the exceptional quality factor of dielectric micro cavities such as glass microspheres and the strong localization of electromagnetic fields by metal NPs such as gold nanorods. The new class of optoplasmonic WGM sensors provide very high detection sensitivity in biosensing. They have enabled the detection of the smallest chemical species in solution, that is single atomic ions [46]. More recently, they have been used to study the dynamics of biomolecular reactions catalysed by enzymes such as the maltose-inducible α-glucosidase (MalL), revealing the MalL conformational state transitions and a negative activation heat capacity [47]. WGM optoplasmonic sensors have been used to study reactions of small molecules on the gold NP surface [47, 48], such as the reversible disulfide reactions and interactions of oligonucleotides and agrochemicals. These various demonstrations have promising healthcare and environmental sensing applications, especially for WGM optoplasmonic sensing integrated with microfluidics on sensor chips [49].

The plasmonic nanorod is the key element of the optoplasmonic WGM sensor that provides single-molecule detection sensitivity. Essentially, the optoplasmonic sensors can be considered single-molecule LSPR (localised SPR) sensors [50, 51]. The plasmon resonance-based sensing at the single-molecule level opens up exciting opportunities for investigating the effects of the localised near field on the chemical reaction [52], improving the limits of detection with plasmonic nanostructures with strong near field enhancements, investigating the ligands interactions and ligand exchange on the plasmonic NPs [48], finding ways for the selective immobilisation of analyte molecules to the plasmonic hotspots, and new sensing modalities that require strong scattering of the NPs [51, 53]or plasmonic heating effects such as thermo-optoplasmonic (TOP) sensing. In some cases, interesting photochemistry can be unravelled, for example by observing DNA hybridization kinetics on the gold nanorods [54].

It has been demonstrated that intense enhanced field confinement (hot spot) on the NP surfaces can be achieved by the introduction of nanoscale features such as spikes to the surface of NPs. Hence, there is a surge in research on the synthesis of metal NPs with sharp tips, corners, and edges (figure 6(B)). The optoplasmonic sensors are currently optimised by choice of plasmonic NP/structure [51], fabrication of high-quality microcavities [53], microfluidic integration which can improve sample delivery and reduce fluidic and thermal sources of noise [55, 56] and by operating the devices at their fundamental detection limits. When laser shot noise dominates, the precision of optical measurements using classical light is limited by the quantum noise limit (QNL) [57, 58]. To date, single-molecule detection operating at the QNL has been demonstrated using a tapered nanofiber sensor with a dark field heterodyne measurement [59]. However, it is possible to surpass the QNL at a given optical power by using quantum correlated light sources, for example, entangled photon pairs and squeezed light. As an example of how quantum optics can be applied to measurements using WGM micro resonators, Li *et al* [60] presented a magnetic field sensor with a 20% improvement in sensitivity by using squeezed light.

### Current and future challenges

The coherent collective oscillation of free electrons in metal NPs may decay either radiatively in light or non-radiatively, producing high energy electron-hole pairs, termed hot carriers. These hot carriers may relax via electron-phonon coupling locally heating the particle or reach the particle surface and transition into unoccupied levels of acceptor adsorbates and trigger chemical reactions. Investigating the photochemistry on the optoplasmonic WGM sensors represents one of the challenges and potentially highly promising fields of study in hot carrier technologies, where hot carriers can catalyse chemical reactions by interacting with external molecules at the particle surface (figure 6(B)) [61, 62]. This includes the degradation of organic pollutants from wastewater, hydrogen generation by solar water splitting and reduction of CO_2_, amongst others. The photocatalytic efficiency depends on various factors, such as hot carrier generation rate, hot carrier energy distribution, rate of adsorption of molecules and chemical stability of the photocatalyst.

One future challenge is combining single-molecule sensors with quantum technology which promises to be the next great leap in optical sensing technology. Quantum optical measurements and sensing with WGMs promise to make this leap happen. The future challenge is to explore the sensitivity limits of WGM single molecule sensors. If the quantum noise limited regime can be reached [59], then the sensitivity could potentially be improved even further by applying entangled photons and squeezed light, as demonstrated in [60]. Existing measurement schemes from quantum metrology will guide these efforts [57, 58].

An improved signal to noise ratio in WGM signals would mean higher confidence in the detection of small molecules, but also could reveal more information present in transient interaction signals. Measurements exploiting quantum correlations are promising for developing highly non-invasive sensors by achieving better sensitivity than that given by the QNL, at low optical powers. These could find applications in the study of photosensitive samples highly prone to photodamage. These next-generation single-molecule sensors will deliver revolutionary advances in our ability to detect biomolecules, investigate, and exploit their complex quantum chemistry.

### Advances in science and technology to meet challenges

Interesting chemical systems are starting to emerge that are investigated on plasmonic NPs and that lend themselves to WGM sensing. Xu *et al* [63] investigated the mechanism of surface plasmon‐assisted catalysis reactions of pATP (p-aminothiophenol) to and back from DMAB (p,p′-dimercaptoazobenzene) on a single Ag microsphere under an atmosphere containing O_2_ and H_2_O vapour (figure 6(B)). pATP was converted into DMAB due to the energy transfer (plasmonic heating) from SPR to the surface‐adsorbed pATP. Under this condition, oxygen, which acts as an electron acceptor, was essential for the conversion reaction, and H_2_O, which acts as a deprotonation agent, accelerated the reaction. On the other hand, the presence of H_2_O, acting as a hydrogen source, induced the hot electron‐promoted reverse reaction (conversion of DMAB to pATP). Further, the photocatalytic oxidation reaction (conversion of pATP to DMAB) is strongly dependent on pH value. The inclusion of secondary metals such as Pt or Pd into the gold nanorods can greatly enhance their photocatalytic performance compared to the individual components [64]. For example, Majima *et al* demonstrated the plasmon-enhanced catalytic formic acid dehydrogenation on Pd-tipped Au nanorods at lower temperatures.

To achieve the goals in quantum sensing, first, we must establish WGM sensors operating at the QNL by using techniques such as homodyne detection, and methods to limit or compensate for thermal noise. Then, by developing measurement schemes using entangled photon pairs or quadrature squeezed states with WGM resonators, the QNL could potentially be surpassed for single-molecule detection (figure 6(C)). Entangled states such as N00N states can improve phase resolution in an interferometer beyond the QNL, while squeezed states of light may be used to reduce the phase or intensity noise of a measurement below the shot noise level, depending on the type of squeezed state generated [57, 58].

Integrating components into on-chip devices could significantly reduce the complexity of the setups required in the long term. Indeed, using recent advances in integrated quantum optical devices, on-chip quantum optical biosensors could be an exciting future area of research [57].

This is the right time to develop quantum sensing on WGM sensors. The single-molecule sensing capabilities of the optoplasmonic sensors will leapfrog with the application of quantum optics techniques and quantum technologies. Similar to the large-scale LIGO interferometer, where the application of quantum optics allowed the investigation of gravitational waves on the km-scale, applying quantum optics to micron-scale single-molecule sensors will allow the exploration of quantum biology & chemistry of molecules on the nanometre-scale. Quantum-optical WGM sensors will deliver unparalleled capabilities for sensing single molecules at the relevant length and timescales, surpassing classical limits of optical detection and unravelling new quantum phenomena. The sensors will offer insights into the quantum properties of living matter.

### Concluding remarks

Although numerous researchers have contributed to the progress in hot electron‐induced chemical reactions, offering potentially high chemical reaction efficiency, this field is still in its infancy in terms of practical applications. WGM sensing of photocatalytic processes and at very high detection sensitivities (one electron turnover) may provide scientific breakthroughs in plasmonics and chemistry and open a new era of practical utilization of hot electrons in various chemical reaction systems.

Researchers will apply the quantum optical biomolecular WGM sensors to reveal the fundamental, quantum properties of key biomolecular systems and prepare the ground for their future exploitation. WGM sensors may well uncover and control the quantum properties of i.e. fast-acting enzymes, photochemical reactions that produce key metabolites, and of magneto-sensitive neuroproteins. These breakthroughs will have numerous and varied applications, for example in ultrasensitive sensing for better health and environment, and in novel brain sensing and intervention methods.

## 6. Laser based sensors

### Peter D Dragic

University of Illinois at Urbana-Champaign, United States of America

### Status

It is often anecdotally stated that the laser appeared as a ‘solution seeking problems’ [65]. The naysayers promoting this viewpoint at that time were not witness to the technological struggles of the light-based sensing systems of the era. For example, the very first pulsed optical rangefinders were developed as early as the 1930’s [66]. However, progress in this area stagnated partly due to a lack of appropriate light sources, namely those with sufficient brightness. Within just a few years following Maiman’s demonstration of the first ruby laser in 1960, the world was witness to the first uranium, HeNe, Nd:Glass, GaAs (semiconductor), YAG, Xe, Ar^+^, CO_2_, chemical, and dye lasers, among many others. In addition to continuous-wave (CW) lasers, Q-switching and mode-locking were also demonstrated within these golden years. This yielded a flood of new light sources that produced a wide range of colours, power levels, and beam characteristics. For those who already understood the ‘problems’ these lasers could address, the experience (or technological leaps) over those few years must have been much akin to handing someone a blow torch soon after they first learn how to start a campfire.

**Figure 7.**
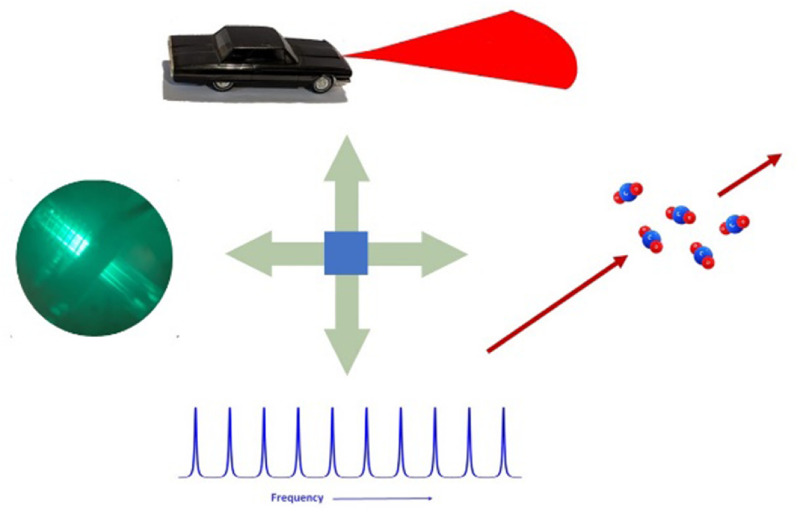
A few examples of laser-based sensing. Going clockwise from the top, automotive lidar, greenhouse gas and chemical sensing, frequency comb-based sensing, and spectroscopy and quantum.

Moving into the modern era, lasers and optical remote sensing are inextricably coupled, and likely always will be. There is, therefore, no further need to justify the purpose of this section: to promote continued advancements in laser development for lightwave sensing applications. The previous Roadmap put forward a definition for laser-based sensors as those that ‘are distinguished from other optical sensors from the perspective that the measurement is entirely based upon the direct detection of laser light itself without relying on any external signal-transducing elements to the target object besides its ambient medium [14].’ Adopting this framework, it is the design and engineering of novel lasers, their capabilities and characteristics that will bring about improvements in system sensitivity, resolution, and accuracy. Laser-based sensing applications (figure 7) include ellipsometers, imaging, spectroscopy, vibrometry, interferometry, lidar, and quantum [67]. Each comes with its own set of system and resulting laser requirements that must be carefully identified by the user. While laser-based sensing systems may be static or on a moving platform, on the ground or in space, use just a few photons or require high power, laser design is always at the forefront, yet never without considering the detector properties that will be needed for the task (e.g. responsivity curve, sensitivity, SNR, etc).

### Current and future challenges

**Figure 8.**
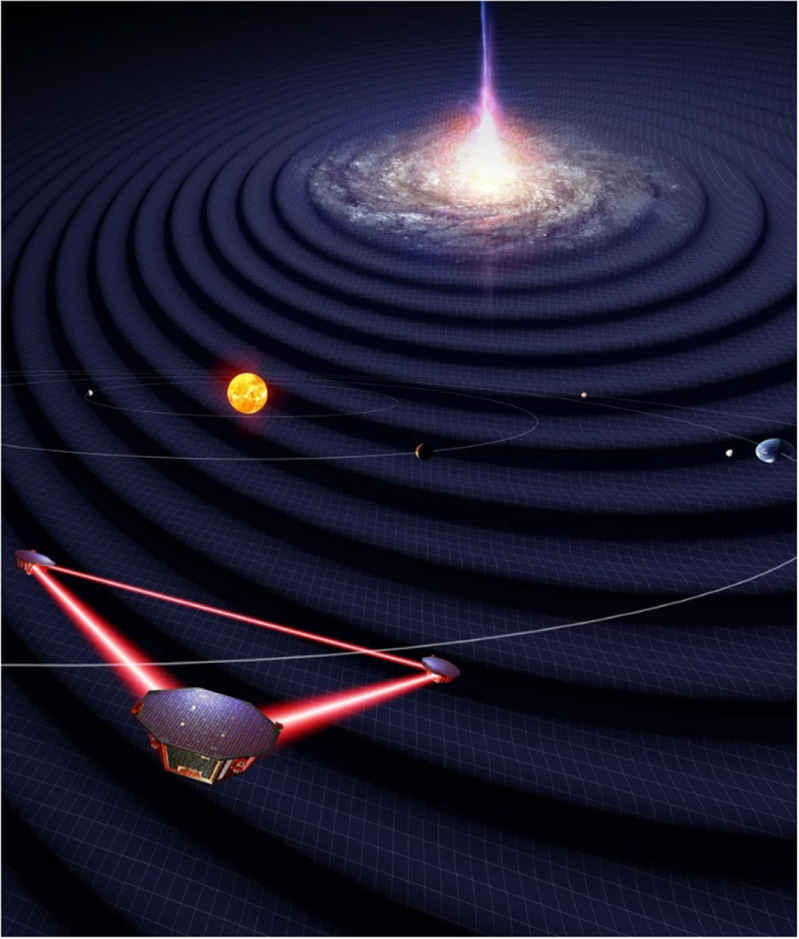
Rendering of the Laser Interferometer Space Antenna actively measuring gravitational waves from, for instance, an extreme mass ratio inspiral, by Simon Barke/University of Florida. Reproduced from Max-Planck-Gesellschaft/Albert Einstein Institute. © University of Florida/Simon Barke. CC BY 4.0.

No existing laser can serve every known sensing application. The complexity in deciding whether a source is appropriate lies in the many attributes, both optical and mechanical (and otherwise), that require careful consideration [68]. These are identified and optimized more or less *à la carte*. Several of these are listed below in the context of laser-based sensing. As an illustrative example, the Laser Interferometer Space Antenna project led by ESA as depicted in figure 8, has strict requirements on both laser coherence, phase noise, and opto-mechanical stability [69].

Laser wavelength. Typical spectroscopic systems, such as the laser detection of gases, require that the laser wavelength be tuned to or across an absorption feature and be stable to well below 1 pm over the measurement time. Often, this requires locking the laser wavelength to an external reference, such as a gas cell.

Laser spectrum. Coherent and spectroscopic systems require single frequency operation with sufficiently narrow linewidth. In the case of spectroscopic systems, most important is the spectrum associated with that to be measured. For coherent systems, the spectrum is driven by the desired coherence length.

Intensity and phase noise. Power instability, laser relative intensity noise, and phase noise further degrade the SNR. There is a growing need to understand the origins of these noises and how to suppress them. This often includes frequency ranges from the millihertz to beyond gigahertz.

Pulse characteristics. Time-of-flight systems, and often those that measure a dynamic quantity, require that the laser be pulsed. The pulse width may set the resolution of the system while the pulse repetition frequency may be set to prevent measurement aliasing.

Beam characteristics. This relates to whether the beam is diffraction limited or not, its divergence, and pointing stability. These characteristics will be particularly important as there is a renewed push into space. As an example, beam expansion relative to a distant receiver geometry reduces divergence and offsets pointing jitter, but in trade can reduce the received power.

Power and energy. This is related to system SNR which in the shot noise limit is proportional to the square root of the number of received photons.

Laser efficiency and temperature. In the quantum limit, the highest possible laser efficiency is related to the quantum defect (QD) which, for optically pumped systems, is defined to be QD = 1–*λ_p_*/*λ_s_* where *λ_s_* and *λ_p_* are the signal and pump wavelengths, respectively. The QD also represents the minimum heat generated in a laser. Laser efficiency and thermal management are particularly important concerns in autonomous and space systems.

**Figure 9.**
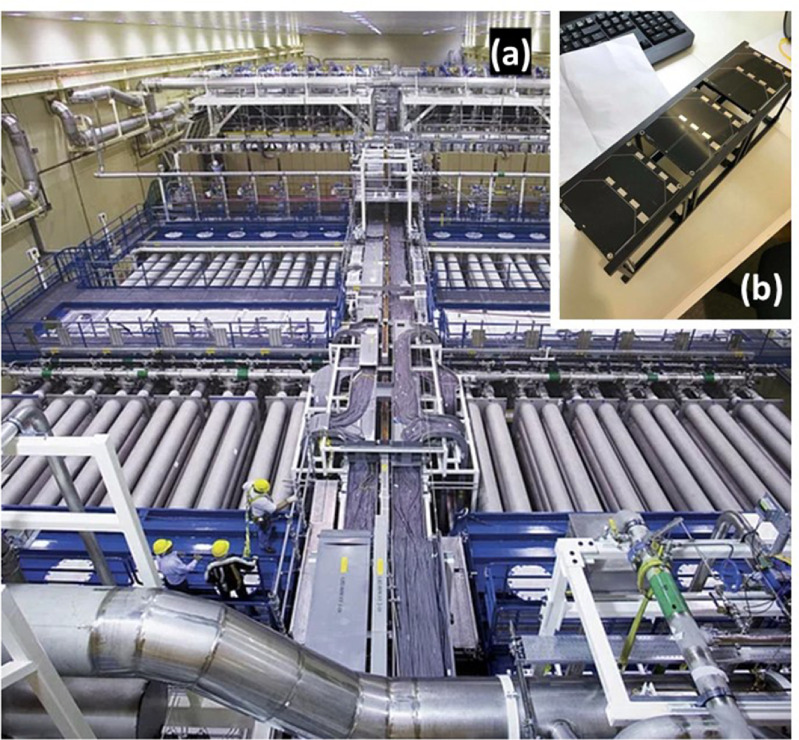
Two lasers of very different scale: (a) The 48 beamlines comprising the United States National Ignition Facility (‘Seen from above’ by U.S. Department of Energy is licensed under CC0 1.0.) and (b) a typical 3U cubesat (30 cm × 10 cm × 10 cm) into which a laser may be installed. A computer keyboard is visible as a point of reference (‘3U Cubesat with solar panel PCBs mounted’ by AphelionOrbitals is licensed under CC BY 2.0.). U.S. Department of Energy via Rawpixel.com / Image is stated to be in the public domain; Aphelion Orbitals via flickr.com / CC BY 2.0.

Thermomechanical environment and SWAP (Size, Weight, and Power). Environmental influences such as temperature and vibration, etc, can introduce deleterious noises that impact system resolution. Space systems may have the additional need to minimize the impact of external radiation damage. SWAP requirements constantly push towards smaller, more efficient lasers. Figure 9 shows laser platforms on vastly different spatial and power scales.

### Advances in science and technology to meet challenges

Lasers are continuously evolving to serve the new and emerging applications that need them. At the forefront of many relatively new areas for lasers, such as lidars for vehicles including self-driving cars, commercial airlines, and spacecraft, is their common call for the laser to be as wall-plug efficient as possible [70]. This could relate to the QD and the generation of heat, or quantum efficiency. In some cases, the laser may have to be cooled on mobile platforms where convective systems such as flowing water may be far too impractical. The use of anti-Stokes fluorescence can enable lasers that are self-cooling [71]. This will assist in the management of thermal noises in lasers whose phases must be tightly controlled. In addition to power (which also reflects efficiency) size and weight budgets drive towards smaller and smaller values. As far as optical properties go, beam geometry and spatial coherence are becoming increasingly important. For example, lasers with structured light beams [72] and random lasers for speckle-free imaging [73] are enabling new sensing modalities with greatly improved performance. Regarding spectrum, there is a need for lasers, both pulsed and CW, producing wider wavelength ranges in the vacuum UV to UV, and mid-to-far IR and into the THz range. This is partly due to the abundance of molecular and atomic absorption features that can be used to probe the various levels of our atmosphere as it relates to pollution, greenhouse gas emission, climate change issues, and space weather. For reasons of SNR, the laser spectrum may be narrower, have the same width, or be broader than the spectrum to be measured. Therefore, on-the-fly control of the power spectral density, such as via phase modulated systems are needed to adapt to the changing conditions of the measurand.

Two final comments should be made here regarding lasers. It is already widely understood that a great deal of mechanical engineering must go into laser design, in particular for those that are meant to be portable. New paradigms in making lasers completely immune to their environments (e.g. vibrations, changes in temperature, etc) will be needed to support the relevant applications, namely autonomous platforms [74]. The final consideration outlined here relates to the complexity in assembling and integrating the laser. Lasers that are more compatible with mass production, are self-aligning, and come with simplified, perhaps modularized redundancies, maintenance and repair will likely win the commercial race to the emerging markets.

### Concluding remarks

The development of lasers and laser-based sensing share a parallel timeline. The former enables the latter, and until the relevant applications become obsolete or unnecessary, improvements to laser technologies will continue to drive system progress. Herein, current and future challenges, as they relate to laser properties and needed advancements in their science and technology, have been briefly outlined. A blossoming new frontier in lasers is their use in space-based applications, including ranging, docking support, interferometric sensors, space weather, and even lidars that can measure compositional characteristics of celestial bodies within our own solar system and beyond. These will require high-efficiency, wavelength-versatile, well controlled beam and low-SWAP lasers that can survive the harsh environment of space.

### Acknowledgments

The author gratefully acknowledges funding from the U.S. Department of Defense Energy Joint Transition Office (DE JTO) (N00014-17-1-2546) and Air Force Office of Scientific Research (FA9550-16-1-0383).

## 7. Mid-infrared sensing

### Ori Henderson-Sapir^1,2,3^ and David J Ottaway^1,2^

^1^Department of Physics and Institute of Photonics and Advanced Sensing, The University of Adelaide, SA, Australia

^2^OzGrav, University of Adelaide, Adelaide, SA, Australia

^3^Mirage Photonics, Oaklands Park, SA, Australia

### Status

**Figure 10.**
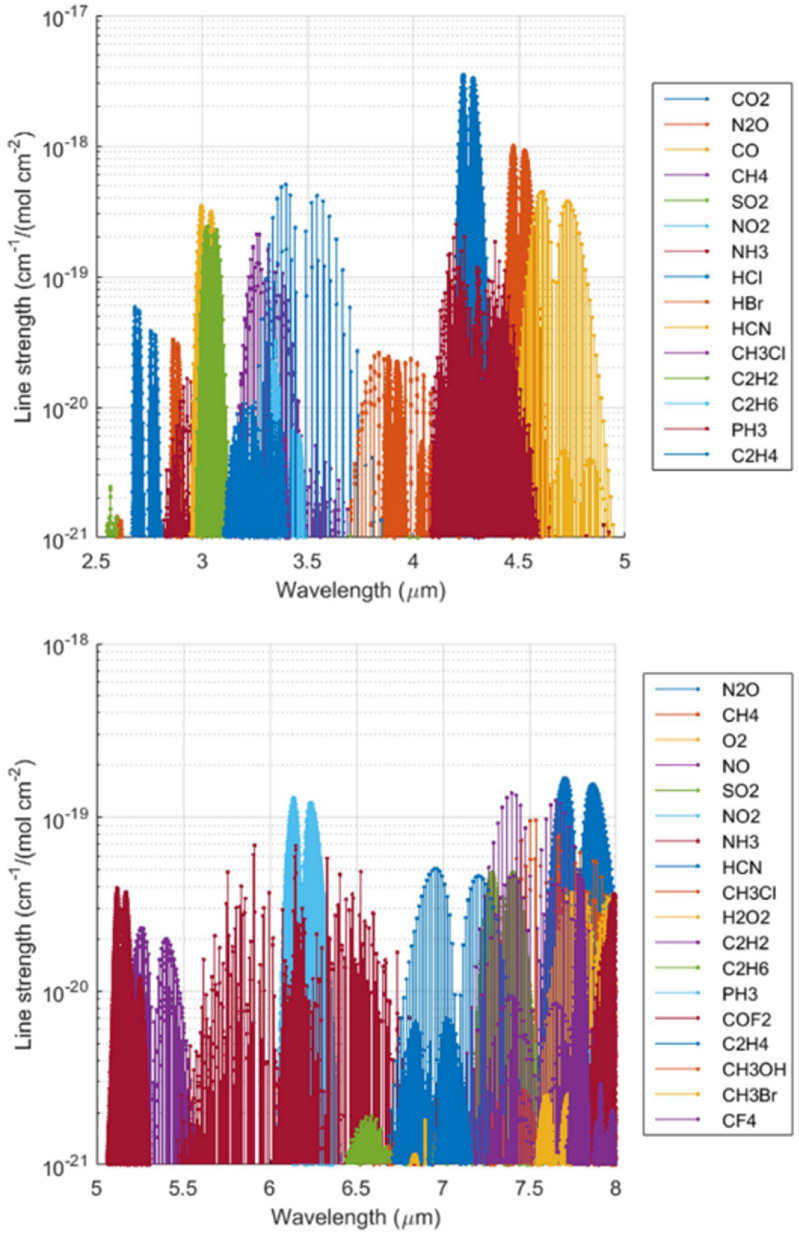
The mid-IR contains fundamental absorption lines for trace gasses. Data from www.spectralcalc.com.

The electromagnetic wavelength range between 3–8 *µ*m is commonly referred to as the mid-infrared (MIR) and it offers unique opportunities for the remote sensing of trace gas (see figure 10) and volatiles. These include key greenhouse gases like methane and ethane and atmospheric pollutants such as NO*_x_* and SO*_x_*. It also promises improved characterisation and detection of organic substances in various settings. An extraordinary potential exists for MIR based disease and medical conditions detection with breath analysis using an inexpensive platform to be deployed in medical clinics. Despite initial steps in these directions, this holy-grails of MIR sensing has yet to be fully realized.

The advantage that the MIR offers for the aforementioned applications is due to the strong characteristic absorption features present in this band, caused by rotational-vibrational bond excitation. The specific absorption features exhibited in this band are often referred to as the ‘absorption fingerprints’. These MIR absorption features can be two orders of magnitude stronger than their overtones in near-IR, which are currently used for sensing applications. Many of the techniques developed for the near-IR can be applied to the MIR to take advantage of the stronger absorption features present there.

The sensitivity of an active sensing platform is governed by the properties of its illuminating light source and detection system. In previous decades, the availability of convenient sources of coherent radiation in the MIR has been limited but there has been significant source development in recent years. Development in MIR detectors has occurred such that the detectors performance is approaching the limit set by thermal background radiation over much of the band.

MIR sensing applications are becoming more ubiquitous as visible and near-IR sensing techniques have bridged the gap to the MIR. Two notable examples are the usage of silica-glass based hollow core fibres and dual-comb spectroscopy. The latter is extremely promising for revolutionizing MIR sensing once MIR frequency combs in convenient and robust packages become available commercially, because at the moment they are still in their infancy.

### Current and future challenges

The signal to noise ratio for the detection of greenhouse gasses and volatiles increases with the spectral brightness of the illuminating source, the collection area of the primary detection optic and the measurement time. Convenient MIR sources with high spectral purity, excellent beam quality and reliability must be developed to take MIR sensing from the lab to the field.

To increase the sensitivity of in-situ measurements, interaction length is often a key parameter. Microstructure fibres such as suspended or hollow core fibres allow light to interact with a trace gas over extended distances with small volumes. Much work is needed to translate these technologies that are mature in the near IR to the MIR. New MIR frequency comb sources will allow the revolution in parallel sensing in the near IR to be translated to the MIR.

Broadband ‘white light’ sources based on supercontinuum generation now routinely cover the entire MIR band and are becoming commercially available thereby making significant inroads especially in spectroscopy applications [74]. However, some applications call for the unique spectral purity and stability that frequency combs offer. Recently, there have been various MIR frequency comb sources demonstrated, but significantly more work is required to turn them into robust turn key sources [75].

Quartz-enhanced Photo acoustic spectroscopy techniques have been employed as an effective method for sensing of trace concentration of greenhouse gasses and volatiles [76]. Detection levels down to the parts per billion and even part per trillion were achieved using this mature detection technique. Even lower detection limits are possible with potentially higher power sources at the short end of the MIR.

Many challenges remain when working in the MIR. First and foremost is the lack of fibre-based components. Since the previous review, a few MIR based fibre components have been demonstrated in the literature suggesting a change in the trend, however, none have become commercially available. We expect a proliferation of MIR fibre lasers and sensing applications as components become commercially available. Therefore, creating a new MIR revolution, similar or even surpassing in magnitude the one that was enabled by availability of near-IR components.

### Advances in science and technology to meet challenges

Significant advances in light sources and delivery are needed to realize the full potential of the MIR, these areas dominate the following discussion.

Interband and Quantum cascade laser diodes (ICLs and QCLs) cover the 3–6 *µ*m and 3–11 *µ*m bands respectively [77, 78]. Their direct electrical excitation is convenient and currently multi-watt average power levels are available (for QCLs) at room temperature, in a significant fraction of the aforementioned band. Their small footprint provides a convenient platform for integration into compact trace gas sensing devices. Cascade lasers suffer from short upper state lifetimes, typically picoseconds for QCLs and sub-nanosecond for ICLs. This limits energy storage making achieving peak power challenging [78]. However, mode-locked operation was recently demonstrated in both QCLs and ICLs [77, 78]. Work is needed to increase their spectral bandwidth and stability for practical comb-based spectroscopy applications.

Near-IR frequency combs have advanced lab-based spectroscopy, metrology and remote sensing. Extending these tools to the MIR will increase sensitivity with ppb sensitivity recently demonstrated [75], utilising the significantly stronger absorption MIR features. MIR micro-resonators generation and frequency shifting of near-infrared combs to the MIR have been demonstrated, including dual-comb methods [79], however, more work is needed to provide convenient and inexpensive comb-based sources in the MIR.

The average power emitted by MIR fibre lasers has increased to 40 W output at 2.7 *μ*m, 10 W at 3.2 *μ*m, 15 W at 3.4 *μ*m and 200 mW at 3.9 *μ*m [80], with increased power and longer wavelength emission an ongoing goal. Fibre lasers have long upper state lifetimes, allowing significant energy storage, making high peak power possible in a compact and rugged device. Ultrafast fibre laser operation has been demonstrated in the short MIR on various bands ranging from 2.7 *μ*m [81] to 3.5 *μ*m band [82]. In Q-switched operation the highest peak powers used the 2.8 *μ*m transition in erbium. Peak powers of 15 kW in a single transverse mode [83] have been demonstrated. Numerical modelling suggests that peak powers approaching 1 kW are possible from the 3.5 *μ*m transition in erbium [84]. Once spectral control is achieved at these peak powers, fibre laser MIR lidars will soon follow.

Pushing the output of fibre lasers significantly beyond 3.5 *µ*m needs lower phonon glasses such as Indium Fluoride, which demonstrated 200 mW average power at 3.9 *µ*m [85]. These glasses reduce absorption losses and non-radiative quenching [85]. Indium fluoride and various chalcogenide glasses are the main contenders for further advances since they have the lowest maximum phonon energy of the semi-mature soft glasses. Indium fluoride is a relatively mechanically robust glass suitable for laser emission in the 4–5 *μ*m range. Chalcogenide glasses have very low maximum phonon energies and the high optical transmission needed for emission beyond 5 *μ*m. However, despite significant effort, only near-IR chalcogenide-based fibres lasers have reported power levels greater than a milliwatt and many challenges remain.

**Figure 11.**
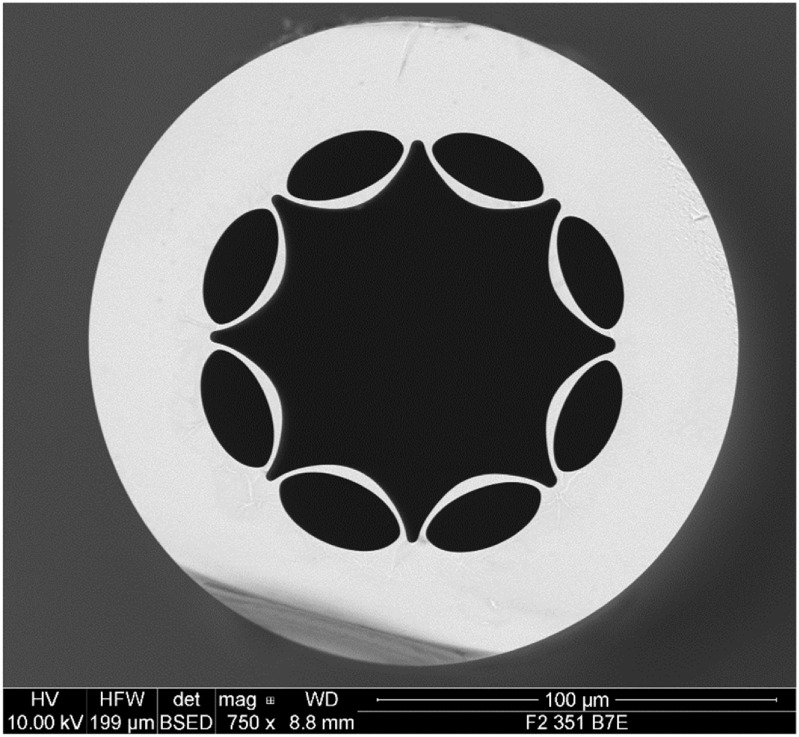
Lead silicate ARHCF developed at the OptoFab node of the Australian ANFF at the University of Adelaide.

Manufacturing micro-structures in MIR transmitting glasses is challenging. Silica-based ARHCF offer an interesting alternative since light is guided within a hollow core with only minimal overlap with silica glass features, see figure 11. ARHCFs have revolutionised guided light delivery and fibre-based sensing in the MIR. The transmission loss of ARHCF fibres now approaches record low-levels for near and MIR transmission. This long interaction length opens the possibility of reaching ppt detection levels of trace gasses in a compact form [86].

Suspended core fibres allow the interaction of the evanescent field with the substance under test. The evanescent field in MIR operation has significantly greater extent which increases the interaction with analyte placed outside the core [87]. Exposed suspended core fibres remove the slow process of filling the fibre. Suspended and exposed core fibres are mature technology for near-IR wavelengths but are yet to be demonstrated in MIR transmitting glasses such as fluorides or chalcogenides.

### Concluding remarks

Development opportunities are abundant in the MIR range for improving trace gas sensing techniques which are highly sensitive as well as selective. The field of MIR sensing benefited significantly from the development and advances in recent years of new light sources. However, for MIR sensing to ultimately fulfil its promise, there is much more yet to be done.

### Acknowledgments

This work was performed, in part, at the Opto Fab node of the Australian National Fabrication Facility supported by the Commonwealth and SA State Government. We thank Dr Erik Schartner for providing the AHRCF image.

This work was supported in part by the US Air Force Office of Scientific Research Award FA-9550-20-1-0160 and an Australian Research Council Discovery Grant 220102516 DP.

## 8. Terahertz sensors and sensing with terahertz

### Elodie Strupiechonski^1^, Goretti G Hernandez-Cardoso^2^, Arturo I Hernandez-Serrano^3^, Francisco J González^4^ and Enrique Castro Camus^2^

^1^CONACYT-CINVESTAV-Queretaro, CIDESI, Mexico

^2^Philipps-Universtät Marburg, Germany

^3^University of Warwick, United Kingdom

^4^Ciacyt-UASLP, Mexico

### Status

Terahertz (THz) radiation is usually defined as the region of the electromagnetic spectrum ranging from the high end of the microwave band to the lower end of the MIR (0.3–30 THz, 100 mm–10 *µ*m, 10–1000 cm^−1^, 1.24–124 meV). Significant advancements toward improving the efficiency of both THz detectors and emitters have been made over the last two decades, yet, there are still challenges and opportunities to be met in this direction. The potential of THz technologies justifies the continued efforts to develop this field as being of crucial relevance to some of the most important modern problems.

THz waves have unique properties which enable non-destructive, non-ionizing, and label-free sensing, facilitating novel applications in imaging and spectroscopy [88]. Because this frequency range has such exciting prospects, terahertz technology has clearly become an emerging field with significant growth on the scientific front. It is now entering the commercial markets for end-users in the healthcare and pharmaceutical industries [89], defence and security [90], non-destructive testing [90, 91], telecommunications, and astronomy.

With the emergence of AI enabled systems and a fast-growing rate of terahertz technologies in research laboratories as well as in the industry, better performing sensors will be critical to the systems’ cost and size reduction and, consequently, to their wider adoption by the end-users for practical applications. Room temperature, low power, low cost, high compactness, and increased sensitivity THz sensors will provide additional and complementary data sets, which are highly desirable for the incorporation of THz technology for non-destructive testing in the real world.

### Current and future challenges

The availability of THz detectors and emitters is the main limitation to the development of THz sensing technologies. THz imaging and spectroscopy systems for sensing can work either in the time domain (THz-TDS) or the frequency domain (THz-FDS). THz-TDS represent the pioneering and most developed systems for coherent, sensitive, and fast detection, which used to be bulky (Ti:sapphire laser) and are now available in reduced dimensions (ultrafast fibre lasers). THz-FDS also exists for real-time sensing and imaging. The drawback is that high sensitivity (low noise equivalent power, NEP) and/or fast detection can be achieved with cooled sensors, which often are bulky and expensive to operate.

**Figure 12.**
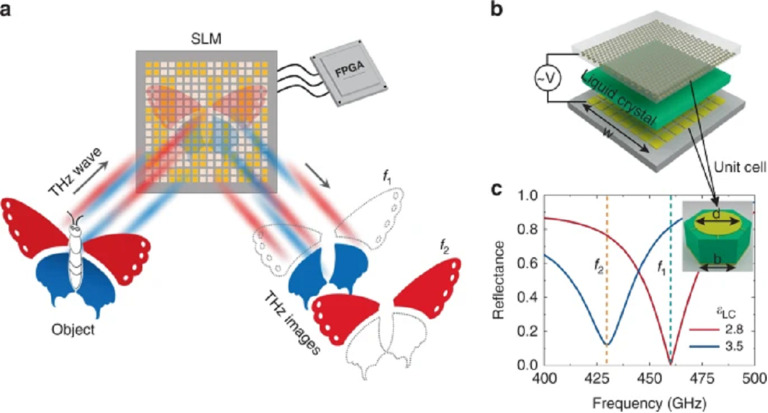
(a) Schematic diagram of the dual-colour THz SLM. (b) Exploded view of the THz SLM. The resonant structures are on the back of the top quartz substrate, and the pixelated gold patches are on the front of the bottom quartz substrate. The thicknesses of the upper and lower quartz substrate are 300 and 500 *μ*m, respectively, the thickness of the LC layer is 10 *μ*m, and the side length of SLM (w) is 19.7 mm. (c) Simulated reflectance spectra of the SLM for different permittivities of LC (ϵLC). The inset is the unit cell of the MMA with *d* = 240 *μ*m and *b* = 173 *μ*m. Reproduced from [96]. CC BY 4.0.

The choice of the best THz sensor essentially depends on the target application. Amongst the most desirable parameters are high sensitivity, broad-band operation, high dynamic range, reliability, high electrical and mechanical stability, and the possibility of being combined into planar arrays. Essentially, the practical needs in terms of detector sensitivity, spectral and spatial resolutions, speed, operation conditions, and space and cost constraints will be decisive in selecting the best THz sensing system for a given application. Within less than ten years, we saw the development of hand-held THz sensing technology [92], real-time near-field THz imaging [93], phase imaging [94], holography, on-chip characterization, THz spectroscopy of biological tissues and liquids [95], and metrology. For the most recent experimental demonstrations, the trend seems to be to modify commercial detector/emitter systems and endow them with new sensing functions to reach enhanced sensitivity or resolution using elements from plasmonics, metamaterials, 2D materials, nanowires, nanoplasmonic fibre tip probes, photonic crystal fibres, microstructured waveguides, or two-channel parallel-plate waveguides. Super-resolution and ultra-compact THz sensors have also been demonstrated by rescaling existing techniques or proposing novel sensing schemes such as single-pixel imaging [96] which is shown in figure 12, total internal reflection with photomodulation [97], or on-chip systems [98]. This indicates that terahertz sensing technologies, within only two and a half decades, are already halfway on the scale of technology readiness [99].

### Advances in science and technology to meet challenges

The THz-sensing application fields that will see the most critical growth are probably the healthcare and the pharmaceutical sector (HPS), followed by nondestructive testing for the aerospace, aeronautics, automotive, and plastics industries. However, for biosensors for the HPS, compliance with regulations is mandatory, and growth may be slowed down on the path from the validation of the devices to the acceptance from the local medical personnel. On the other hand, to meet industry standards, THz sensing industry will need to move towards small size, weight, power, and cost (SWaP-c) sensors. Fast detectors that can be developed in plane-array are desirable for real-time applications where single-pixel imaging cannot be implemented. Since real-time imaging generates large amounts of data at a fast rate, AI will be necessary to incorporate into the THz sensing systems to automate complex pattern recognition and image interpretation. In general, the development of functional THz sensing systems will require a holistic strategy, one that crosses the boundaries of many emerging research fields.

### Concluding remarks

Considering the essential findings and capability gaps briefly described in the previous sections, we identified an increasing demand for terahertz sensing systems in the medical sector and non-destructive testing applications. Applications in security and telecommunications are also critical and will continue to grow. A transdisciplinary strategy, in parallel with recognizing and responding to the lack of awareness of the terahertz technology in general by potential users, is also essential at this stage of the development of this technology.

### Acknowledgments

All the authors of this section are either current or former members of the Laboratorio Nacional de Ciencia y Tecnología de Terahertz (Mexico) and would like to acknowledge the support from CONACYT through various grants. E C C would like to acknowledge the support from the Alexander von Humboldt Foundation.

## 9. Biomedical optical sensors

### Alexis Méndez^1^ and Paola Saccomandi^2^

^1^MCH Engineering LLC, United States of America

^2^Department of Mechanical Engineering, Politecnico di Milano, Italy

### Status

With a growing global population requiring healthcare and the need for ever more sophisticated diagnostic tools, clinicians worldwide are focusing on the use of advanced biomedical instrumentation and sensors as necessary and effective tools for patient diagnosis, monitoring, treatment and overall care. Many of the medical instruments in use today rely on optics (and optical components) to perform their function, and with the development of semiconductor lasers in the 1960s, modern medical optics began to take shape and coupled with the availability of optical fibres, a new generation of optical bio-medical instruments, sensors and techniques began to be developed. The advantages of optical fibres have been recognized by the medical community long ago [100]. Their initial and still most successful biomedical application has been in the field of endoscopic imaging, with the first fibre optic endoscope demonstrated in 1957 [101]. Then, during the 1980s and 90 s, extensive research was conducted to develop fibre-based biological, chemical and medical sensors [102]. Fibre-optic based sensors are ideally suited for a broad variety of—invasive and non-invasive—applications in life sciences, clinical research, medical monitoring and diagnostics, such as OCT probes, force- and shape sensing catheters in robotic surgery, intra-aortic pressure probes and temperature monitoring for thermal-based therapies for localized tumours [103].

To date, biomedical sensors based on external cavity Fabry-Perot interferometers, FBG, and spectroscopic types based on light absorption and fluorescence, are among the most researched and developed into commercial products [104]. Fibreoptic biomedical sensors often rely on the use of special coatings or small cavities holding a specific reagent that can detect a given bio-chemical analyte of interest. This is a common practice in the so-called optrodes, as well as in the use of tilted FBGs. Besides fibre-based devices, integrated optic planar devices are also an attractive and effective platform to develop biomedical sensors

**Figure 13.**
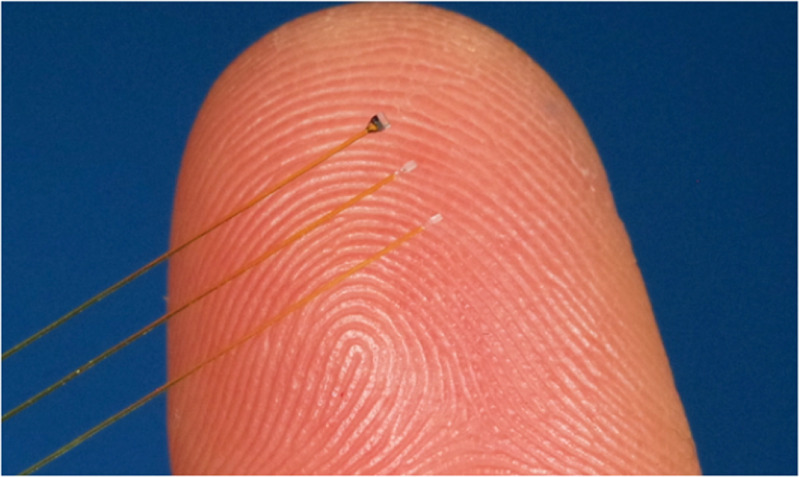
Aspect of miniature fibre optic Fabry-Perot biomedical pressure sensors Reproduced with permission of Resonetics, (formerly FISO Technologies). © Resonetics (Formerly FISO Technologies).

Biomedical optical sensors can be categorized into four main types: *physical, chemical, biological and imaging*. Physical sensors measure a broad variety of physiological parameters such as body temperature, blood pressure (figure 13), respiration, heart rate, blood flow, muscle displacement, cerebral activity, etc. Chemical sensors rely on fluorescence, spectroscopic and indicator techniques to measure and identify the presence of particular chemical compounds and metabolic variables (pH, blood oxygen, glucose). Chemical sensors detect specific chemical species for diagnostic purposes, as well as monitoring the body’s chemical reactions and activity. Biological sensors tend to be more complex and rely on biologic recognition reactions—such as enzyme substrate, antigen-antibody, or ligand-receptor—to identify and quantify specific biochemical molecules of interest. Imaging sensors encompass both endoscope devices for internal observation and imaging, as well as more advanced techniques such as OCT, photoacoustic imaging and others, where internal scans and visualization can be made non-intrusively.

Biomedical sensors also present some unique design challenges. Sensors need to be safe, reliable, highly stable, biocompatible, amenable to sterilization and autoclaving, not prone to biologic rejection, and not require calibration or at least maintain it for prolonged times. In particular, sensor packaging is a critical aspect. It is highly desirable that sensors be as small as possible—particularly those for implanting or indwelling purposes.

### Current and future challenges

Nowadays medical personnel are more reliant on advanced biomedical instrumentation and sensors as tools for patient diagnosis, monitoring, treatment and care. There is also a need for analytical instruments that can provide faster results on blood and other sample analyses, which can facilitate on-the-spot actionable diagnosis. In addition, advances in minimally invasive surgery coupled with the advent of medical robotics and computer-assisted surgical systems, is demanding the development of smaller disposable sensing catheters and sensing probes. These needs are offering many opportunities for the design and development of optical sensors but besides ensuring that the devices are safe, effective, easy to use, fast-responding, low-cost, there is also the challenge of identifying a suitable sensing technique and a platform that can be exploited for multi-parameter sensing.

### Advances in science and technology to meet challenges

Among numerous innovations taking place in the fields of optics and photonics, we can identify four key breakthrough technologies that hold great potential for biomedical sensing. The first is Raman spectroscopy. Spontaneous Raman scattering is a result of inelastic light scattering processes, which lead to the emission of scattered light with a different frequency associated with molecular vibrations of the identified molecule. Several techniques have been developed to enhance the signal, such as coherent anti-Stokes Raman spectroscopy, stimulated Raman spectroscopy (SRS), resonance Raman spectroscopy, and SERS, all becoming prominent techniques for optical biosensors and bioimaging. Advances in pulsed laser sources have allowed exploring vibrational features of biological structures. Hence, SRS is commonly used as a probing technique for ultrafast and time-resolved characterization of biological systems, such as myoglobin [105]. In SERS, the inelastic light scattering by molecules is strongly enhanced (by factors up to 10^15^, reaching the single molecule level) when the molecules are adsorbed onto specific substrates of corrugated metal surfaces embedding metal (silver or gold) NPs [106]. The selective detection and localization of target molecules requires target-specific ligands for molecular recognition via non-covalent interactions, the nanotags. This technology has opened the door to other research fields, with diverse biomedical applications. An example is the development of theranostic platforms based on gold NPs which combine SERS detection for *in vivo* cancer diagnosis and light-based therapies (e.g. photodynamic therapy, photothermal therapy or photoimmunotherapy). Other uses are the combination with microfluidics to perform immuno- and cellular-assays, and the diagnosis of degenerative disorders (e.g. presence of amyloid proteins in Alzheimer’s diseases), infectious diseases (e.g. the presence of virus), or genetic diseases (i.e. presence of mutations in DNA). It is expected that optical SERS-based microfluidic platforms and lab-on-chip will have a substantial impact on biomedical diagnostics in the near future. Novel fibre optic-based sensing platforms are nowadays exploiting SERS for biological measurements. Tip-coated multimode fibre, liquid core photonic crystal fibre and other configurations are employed as the SERS probe for remote and multiplexed detection [107].

Nanophotonic devices, which control light in sub-wavelength volumes and enhance light–matter interactions [108], represent another key innovative technology for biomedical sensing, driven by the synergy between fibre optic sensors and nanotechnology. The advancement of deposition of nanomaterials has given a boost to the area of optical fibre sensors. Nanostructured thin films and nano-coatings, such as gold and graphene, have been applied to several optical fibre configurations for the development of new sensors, including conventional fibres (e.g. etched fibres, or multimode fibres based on SPR and localized SPR), grating-based fibre (e.g. FBGs, tilted FBGs, long period FBGs), and microstructured optical fibre for detecting multiple physical and biochemical parameters [109]. Other novel capabilities brought-on by these devices are in the form of the so-called lab-on-a-fibre (LOF) [110], where functionalized thin layers of micro- and nano-particle materials are deposited on the tip of an optical fibre.

A third driving technical advance, particularly marketwise, is presented by wearable fabrics and clothing for diverse health, wellness, sports and fitness applications. For example, skin-like wearable optical sensor patches with optical nanofibers embedded, are being proposed for continuous monitoring of temperature and respiration parameters [111]. Similarly, smart textiles with woven fibre optic sensors are also under development for monitoring physiological parameters, such as breathing and cardiac rate [112]. Wearables fitted with optical sensors represent one of the milestones towards the realization of an effective personalized medicine [113].

Lastly, small-size and flexible optical fibre sensors are increasingly entering in the design of minimally invasive medical devices such as surgical robots. Technologies based on high-density FBGs or distributed sensing, based on Brillouin and Rayleigh scattering, allow for accurate and spatially resolved information along the entire length of a surgical instrument (pressure, strain, temperature), without the use of additional devices [114].

### Concluding remarks

Optics is a versatile enabling technology for the development of present and future generations of novel biomedical sensors and sensing techniques for diagnostic, therapeutic and surgical applications. Biomedical optical sensors are becoming increasingly pervasive across the medical industry, finding applications in pharma, biotech, as well as in medical wearables and surgical robotics. However, their development is not trivial and proper design, materials selection, bio-compatibility, patient safety and other key issues must be properly considered to pass industry certifications and ensure commercial success.

## 10. Single molecule detection in diagnostic assays

### Qimin Quan^1^ and Zhongcong Xie^2^

^1^NanoMosaic Inc., United States of America

^2^Massachusetts General Hospital and Harvard Medical School, United States of America

### Status

**Figure 14.**
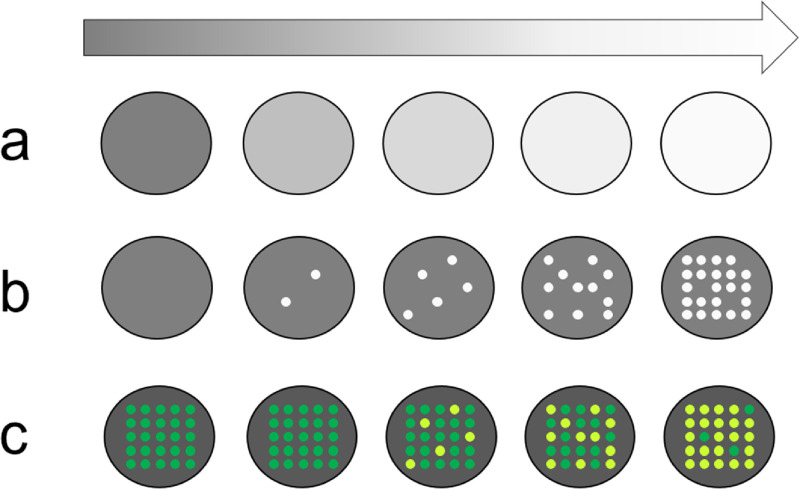
(a) Analog read-out: signal increases as the analyte concentration increase. (b), (c) Digital readout: signal is digitized as it is originated from a single molecule. The read-out can be either light intensity (b) or spectrum shift (c).

It is estimated by The Centres for Disease Control and Prevention (CDC) that 70% of today’s medical decisions are based on the laboratory test. About 200 proteins are cleared or approved by the US Food and Drug Administration (FDA) or approved under the Clinical Laboratory Improvement Amendments (CLIA) regulations, for detecting cardiac, cancer, diabetes, infectious and other diseases [115]. The transition from the model of intervention to prevention is an ongoing effort in the healthcare system, which is a major force that pushes the limit of diagnostic assays for better sensitivity and specificity. Protein biomarkers are exceptionally challenging since no amplification mechanisms are available to increase the copy number of proteins, unlike nucleic acids. The complex sample matrices (such as plasma/serum, cerebrospinal fluid, urine) and wide ranges of concentrations of different proteins require the detection method to have both high sensitivity and large dynamic range. Optical immunoassays are the primary method for protein quantitation and demands improvement on sensitivity and accuracy. The two fundamental steps in immunoassays are (1) capturing target analytes with high selectivity using specific affinity probes, and (2) transducing the binding events into a physical readout that is sensitive and robust to implement. Developing high affinity probes, including antibodies and their fragments, aptamers, engineered molecular constructs is an active and important field, although it is outside of the scope of current discussion. This section will focus on the readout mechanisms and discuss the current challenges and future opportunities of using single molecule optical detection (figure 14) to improve the sensitivity, specificity, and accuracy of diagnostic assays. Technical advances in the readout formats will fit for all types of binding modalities.

### Current and future challenges

#### Limit of quantitation.

Cytokines are the class of proteins of lowest concentrations in the human proteome. An order-of-magnitude analysis of the nominal concentration of cytokines will be useful in determining the limit of quantitation to be reached. For example, there are approximately ∼100–1000 CD4 cells per ml of blood that produce 1000–10 000 proteins per cell, which will be secreted and diluted into 5 l of blood. Thus, cytokine detection requires a limit of quantitation down to the level of 100 fg ml^−1^. Neurological biomarkers are also present at similar concentrations (100 fg ml^−1^–10 pg ml^−1^) in blood since they must cross the brain-blood-barrier. Current gold standard method, enzyme-linked immunosorbent assays (ELISA), typically reaches the lower limit of quantitation at around 10 pg ml^−1^. Fluorescence, chemiluminescence and electrochemiluminescence have shown better detection limit and dynamic range, thanks to the advancement in CMOS and CCD imaging technologies. However, consistent performance below pg ml^−1^ is still challenging.

#### Precision and accuracy.

Both precision (% coefficient of variation, or CV) and accuracy (mean % deviation from nominal concentration) are key parameters to evaluate the analytical performance of an assay. CV of 20% (and 25% at the lower limit of quantitation) is the FDA recommended acceptance criteria for protein assays, while a high accuracy is especially important when applied to discover and validate new biomarkers, since the fold increase (or decrease) in the disease phenotype is in many cases less than 2. Most accurate nucleic quantitation method, digital PCR, has achieved intra-CV of 2% and inter-CV of 5% [116], and is widely used in the manufacture quality control process of gene and cell therapy. It should also be noted that at ultra-low concentrations, molecular shot noise should be considered. For example, to achieve CV of 2% at 1 fM concentration, at least 2500 molecules need to be detected (square root of number of molecules) [117], which puts a fundamental limit to the minimum sample volume at 4 ul.

#### Absolute quantitation.

Current protein assays rely on spiking calibrator proteins into a surrogate buffer to build the standard curve. FDA guidelines recommend that the standard curve buffer should be identical or as similar as possible to the sample (especially for pharmacokinetic applications), a surrogate matrix is often used in assays since no current technology is able to detect every single protein in a given volume, unlike digital PCR. Eliminating the need for calibration curves will simplify the assay workflow, and alleviates the cost burden associated with developing standards, calibrating instruments, and bridging studies.

#### Multiplexing.

FDA cleared or approved (including under the CLIA program) protein biomarkers represent only 1% of the human proteome [115]. Discovery tools such as mass spectrometry can cover a few thousand proteins, still a fraction of the whole proteome. A high multiplex technology without compromising the detection sensitivity and quantitation accuracy may lead to new ways of diagnostics, where a disease phenotype is correlated to a combinatorial indication of large number of biomarkers.

### Advances in science and technology to meet challenges

**Figure 15.**
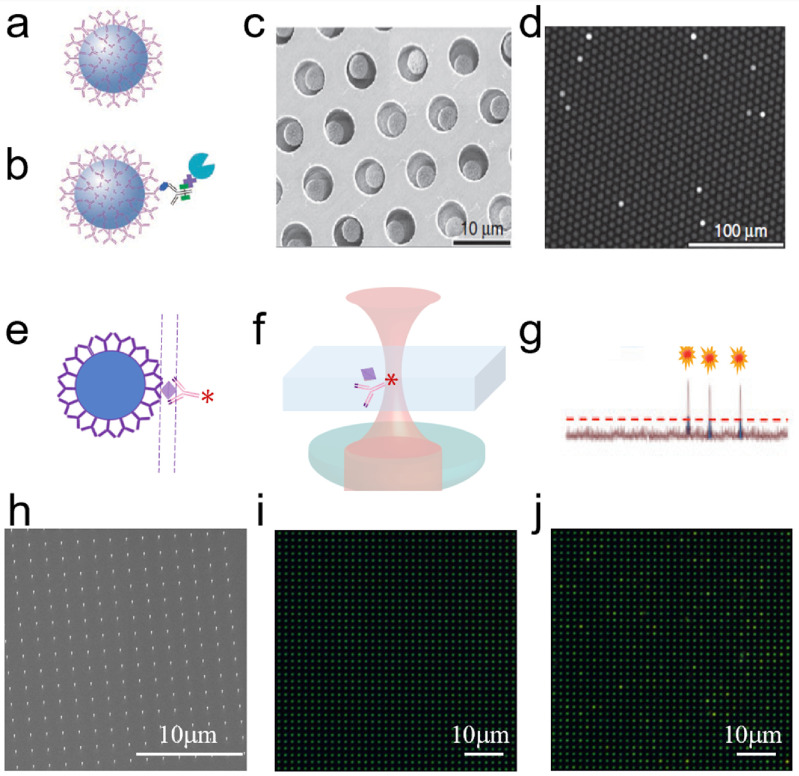
(a)–(d) Single molecule array technology [120]. Bead is coated with capture antibodies (a) and antibody-antigen sandwich complex is formed on bead (b). Beads are dispersed in microwell array (c). Fluorescence signals from enzyme reactions (d) are counted. E-g. Single molecule counting technology [121]. Reproduced from [120], with permission from Springer Nature. Antibody-antigen sandwich complex is formed on bead (e), then eluted to the focus spot of confocal imaging (f). Each fluorescent spike is counted (g). (h)–(j). Nanoneedle technology. Nanoneedles (<100 nm) are fabricated in the array format (Scanning Electron Microscope image in (h)). Antibody-antigen sandwich complex induce a spectrum change of each nanoneedle between the pre-image (i) and post-image (j).

The effort to push the limit of immunoassays traces back to 1970s, when Harris *et al* [118] demonstrated an improvement of 100-fold over ELISA and reached a limit of detection 10^−21^ M by radio-labelling the enzyme substrate and allowing the enzyme reactions to last several hours. Although radioactive biohazards restricted the wide application of radioimmunoassay, this work is important as it demonstrated that improving the readout will significantly improve the assay performance under the same biological conditions. By replacing the radiolabels with fluorescent detection, Rondelez *et al* [119] further demonstrated that confining the enzyme reactions in isolated, micrometre-sized wells will accumulate enough fluorescent signal from a single enzyme molecule. The fluorescence is detectable using conventional microscopy and the enzyme reaction time is reduced to a few minutes. Rissin *et al* [120] further extended the confined enzyme amplification approach and incorporated it into the ELISA workflow. Antibody coated beads are mixed at excess concentrations with the target analyte, that pushes into the Poisson distribution regime that single, or no molecule is bound on each bead. The beads are then loaded into isolated microwells where enzyme reactions are confined (figures 15(a)–(d)). An improvement of >1000 times over ELISA is demonstrated, and this novel single molecule array (Simoa^TM^) approach is coined as digital ELISA. Two other single molecule approaches are commercially available now. The single molecule counting (SMC^TM^) technology replaces enzyme amplification process with confocal imaging of single fluorescent reporters [121]. Similar to the digital ELISA, antibody-analyte sandwiches are formed on beads and the detection antibodies are fluorescently labelled, which are eluted into the focus spot of a confocal microscope and counted for single molecule fluorescent events (figures 15(e)–(g)). The nanoneedle technology (MosaicNeedle^TM^) replaces both beads and fluorescent labels with nanoneedle biosensors that detect the optical spectrum shifts induced by the antibody-analyte complexed formed on the nanoneedles [122, 123]. The nanoneedles are 100-fold smaller than the bead, hence higher multiplexing level can be achieved with significantly smaller footprint (figures 15(h)–(j)).

The prevalence of hydrogen bonds, electrostatic interactions and salt bridges leads to a non-specific binding equilibrium constant at approximately 10^−3^M, while high affinity antibodies have equilibrium constants typically at 10^−12^M. Since high-abundance blood proteins are in the 10^−3^M range, non-specific bindings will occupy a large portion of the binding sites as the analyte concentrations fall into the 10^−15^M range (e.g. cytokines and neuro-markers). Therefore, non-specific bindings are the dominating contributor to the background noise in high-sensitivity assay.

Single molecule methods provide a mechanism to effectively increase the SNR. This is because the signals are collected from a confined area and is binary (zero or one). Ratio of signals from specific binding to that from non-specific binding is constant as the analyte concentration decreases. In contrast, SNR decreases as analyte concentration decreases in convention immunoassays based on an analogue read-out. In addition, blocking reagents and washing steps are also critical to suppress non-specific bindings. Utilizing a second antibody to form a sandwich with the analyte and its primary capture antibody also suppresses signals contributed by non-specific bindings.

Single molecule methods provide a 1:1 conversion between the readout signal and the number of analytes. In principle, calibration curve (a set of signal responses as a function of known analyte concentrations) is no longer required. However, absolute quantitation has yet been demonstrated, since only an unknown percentage of the target analytes are captured and counted. Shirai *et al* [124] designed a nanofluidic channel that confines the assay and detection into a 10^2^ nm chamber, allowing a 100% capturing of analytes as the channels are much smaller than the diffusion length of the analytes during incubation.

### Concluding remarks

Single molecule detection has shown clear advantages in sensitivity, precision and accuracy and has the potential to achieve absolute quantitation without the need for calibrators. Although no digital immunoassays have been FDA cleared or approved at the time of this review, applying them to study neurological biomarkers and cytokines, and to monitor the prognostic change of low-abundance biomarkers are widely adopted in basic and clinical research. It should be noted that the assay development life cycle has many phases including method development, pre-study validation and in-study validation. This article has focused on the method development, although a wholistic development plan and early engagement in regulatory conversations are also key to the success of pushing the diagnostic application from bench to bed.

## 11. Nanoplasmonic optical probes in biological imaging

### Björn M Reinhard

Department of Chemistry and The Photonics Center, Boston University, United States of America

### Status

Noble metal NPs sustain size- and shape-tunable LSPRs throughout the visible and the Near-Infrared that provide unique opportunities for biological imaging. Depending on their size, noble metal NPs provide large scattering or absorption cross-sections that facilitate their use as labels in optical microscopy, as well as in photothermal and photoacoustic imaging [125]. Furthermore, the strong E-field localization associated with LSPR excitation enables signal enhancements of Raman labels through SERS for applications in bioimaging [126]. The superb photophysical properties of noble metal NPs also permit theranostic applications in which the NPs have both diagnostic and therapeutic uses [127, 128].

**Figure 16.**
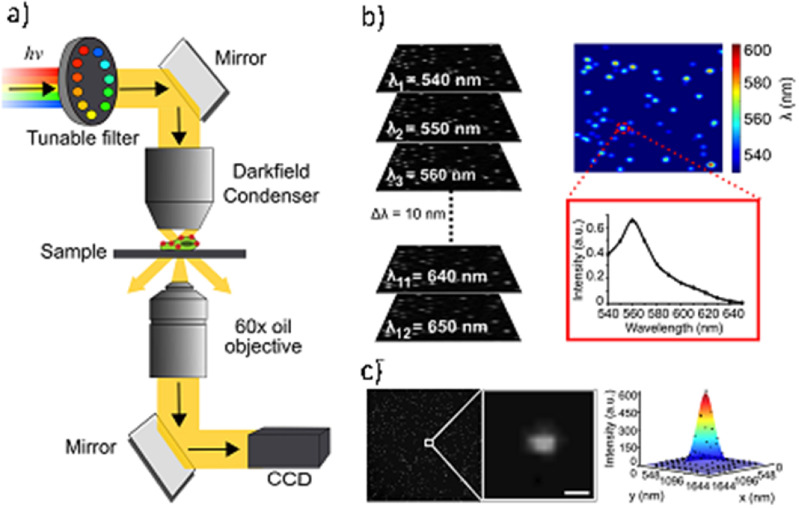
(a) Experimental set-up for hyperspectral PCM. (b) A hyperspectral image of NP probes is generated by recording a set of monochromatic images at defined wavelengths and combining them into a composite image in which each pixel contains a complete spectrum. (c) Field of view containing immobilized gold NPs (diameter ∼ 72 nm), zoom-in of one individual NP, and 2D Gaussian fit. Reprinted with permission from [130]. Copyright (2019) American Chemical Society.

**Figure 17.**
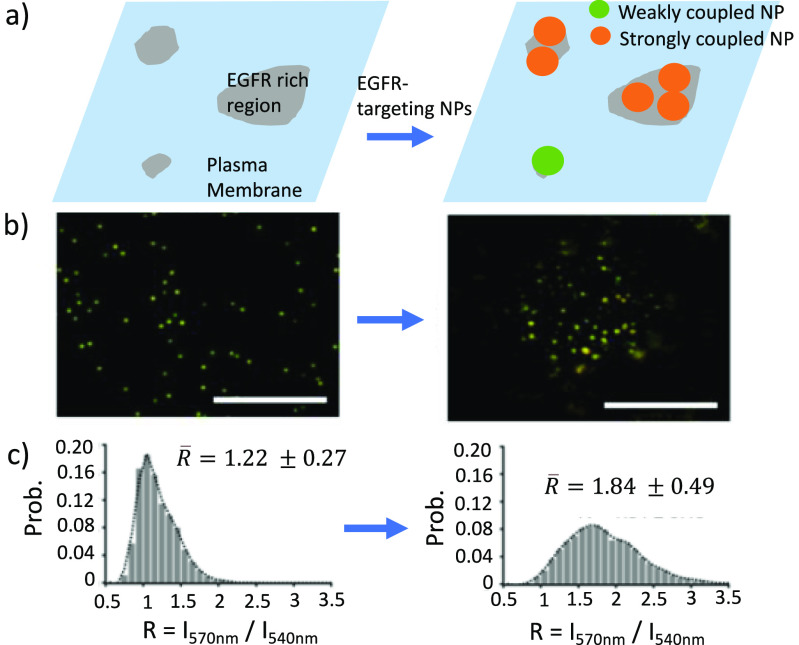
(a) Principle of detecting receptor (here epidermal growth factor, EGFR) clustering in the plasma membrane using PCM. NP binding in close vicinity due to spatial EGFR clustering induces spectral shifts in the plasmon resonance that can be detected in far field microscopy. (b) Darkfield scattering images of 80 nm gold NP labels before (left) and after (right) binding to an EGFR-expressing cell. (c) Colorimetric analysis of the spectral shift induced by NP binding to EGFR. The left histogram shows the distribution of the ratio, R, of the scattering intensities detected from NP labels at 570 nm and 540 nm before binding to EGFR. The right histogram shows the distribution of the intensity ratios after binding. The increase in R indicates a spectral shift in the NP plasmon due to clustering of EGFR-binding NPs. (b), (c) Reprinted (adapted) with permission from [130]. Copyright (2019) American Chemical Society.

A particularly interesting property of noble metal NP probes is that electromagnetic coupling between them shifts the plasmon resonance wavelength. This coupling is distance-dependent and relevant for separations of approximately one NP diameter and below. The spectral shift of the plasmon resonance induced by a close contact between two or more NPs is detectable in the far-field and is exploited in Plasmon coupling microscopy (PCM) (figure 16) to detect sub-diffraction-limit proximities [129]. One important application of PCM is the detection and characterization of the spatial clustering of cell surface receptors. Although PCM does not directly resolve the actual size of cell surface receptor clusters, the resonance wavelength of the NP labels provides a quantitative metric for the spatial clustering of the NP labels, which depends on the spatial distribution of the receptors (figure 17). The ability of PCM to detect and characterize spatial cell surface receptor heterogeneity was evaluated using the fluorescence based super-resolution microscopy direct stochastic optical reconstruction microscopy (dSTORM) as benchmark [130]. The comparative study revealed that the spectral shifts obtained for selected breast cancer cells with different degrees of epidermal growth factor receptor (EGFR) expression by PCM were consistent with differences in average EGFR cluster size as determined by dSTORM. PCM is compatible with high throughput imaging, which makes the technology interesting for screening biological samples and characterizing cell-to-cell variability.

Another important application of nanoplasmonic optical probes is as mimics of virus particles. In artificial virus NPs (AVNs) [131, 132], the metal NP core is encapsulated in a self-assembled hybrid membrane consisting of an inner octadecane thiol layer and an outer self-assembled lipid layer of defined composition. As AVNs consisting of a gold NP and a lipid coating have the surface properties of a biomimetic membrane and the large optical cross-sections of a noble metal NP core, they are interesting biophysical tools for investigating lipid-mediated virus—cell interactions. Specifically, AVNs were used to explore the role of the ganglioside GM3 in the glycoprotein-independent binding of human immunodefiency virus 1 (HIV-1) to CD169 (siglec1) expressing macrophages and dendritic cells and the subsequent intracellular sequestration in non-endolysosomal compartments. GM3-functionalized NPs were also shown to bind to CD169 expressing cells in the lymph nodes of mice after hock injection, facilitating the targeting of these cells in *vivo* [133]. In addition to assembling membranes of defined composition around noble metal NP probes, wrapping of isolated cell membranes around the NP core has been demonstrated to be advantageous for a broad range of *in vivo* applications [134].

### Current and future challenges

The compatibility of noble metal NPs with a wide range of optical imaging modalities, in addition to their large cross-sections in electron and x-ray microscopies, make noble metal NPs versatile labels on the subcellular, cellular, tissue, and whole animal level using different imaging modalities. Unlike fluorescent probes, NPs do not bleach. Due to their multimodality and unique photophysical stability, noble metal NPs are useful probes for applications that require long, continuous illumination or that aim to elucidate the fate of functionalized NPs in biological systems ranging from cells to whole animals. One important trend in this context is the biomimetic design of NPs, such as AVNs, whose size, morphology and surface properties reflect that of biological entities like vesicles or exosomes but whose core contains a noble metal NP with strong optical properties. In these applications, the NPs are more than simply imaging labels. Instead, they represent bio-inspired hybrid materials that combine the surface properties provided by the self-assembled lipid membrane with the optical properties of the noble metal NP core to enable bioimaging and biosensing applications. The development of these technologies requires an exact understanding of the interfacial properties of the NPs and how they are affected by complex biological matrices. Eventually, NP probes with rationally designed surface chemistries in combination with enhanced NP imaging modalities will contribute to elucidating the fundamental mechanisms of NP—cell interactions and show how they affect cellular regulation mechanisms. This gain in knowledge will be instrumental in overcoming challenges associated with developing targeted NP delivery systems and new nanomedicines.

The light-dependent responses of biomimetic NPs with a noble metal core can be exploited to engineer active responses that are missing in biological nanomaterials. It is conceivable, for instance, that the properties of lipid-coated noble metal NPs can be actively modulated by light irradiation, for instance, if the irradiation results in a heating of the NPs that induces a phase change in the membrane, paving a path to adaptable biosensors and bioimaging probes.

In addition to challenges associated with understanding and controlling the interfacial properties of nanoplasmonic probes, the properties of the core also provide opportunities for further improvements. Currently, most applications of nanoplasmonic probes in biosensing and imaging are based on gold and silver NPs, which defines some fundamental limitations for the scope of the approach.

### Advances in science and technology to meet challenges

Improving the control of the interfacial properties of nanoplasmonic probes is challenged by the complexity of the interface that depends on a large number of parameters. However, more efficient molecular dynamics codes and AI algorithms can be expected to advance the accuracy for modelling the interactions of nanoprobes with complex biological matrices. In the future, it may be feasible to determine the surface composition of nanoplasmonic probes for specific applications using appropriate computational tools. Improved modelling capabilities will also help to better understand how the protein corona forms around NPs in biological matrices and how it effects NP—cell interactions.

The last 10 years have seen great interest in the development of new non-metallic plasmonic materials [135, 136], and some of these materials have potential as nanoplasmonic optical probes in biosensing and bioimaging applications. Although the plasmon resonance of gold nanostructures can be tuned over a wide spectral range by adjustment of the size and morphology of the NPs, alternative plasmonic materials provide an additional strategy to control the plasmon wavelength independent of the morphology of the NPs. This is of interest since the fate of NPs in cells or tissue depends on the size and morphology of the probes. To take advantage of alternative plasmonic materials as probes in biological imaging, scalable size and shape-selective fabrication strategies, as well as biocompatible surface passivation approaches need to be advanced.

Technological improvements in hyperspectral imaging and deep learning imaging analysis are expected to further enhance the sensitivity and fidelity of plasmonic NP imaging. In the future, a convergence of different optical NP imaging techniques, including photoacoustic, photothermal, vibrational and scattering based approaches, may result in the imaging of biological processes across multiple length scales in time and space, facilitating the investigation of biological processes from the whole organism down to the cellular level.

### Concluding remarks

Localized plasmons in nanoscale particles give rise to light-dependent responses that are tunable through the morphology of the NPs. These properties make nanoplasmonic probes highly adaptable tools in different imaging modalities. Importantly, the function of the NPs is not limited to that of simple labels, but instead can involve biomimetic or photo-responsive functions that can probe biological systems in a unique way. Therefore, nanoplasmonic probes complement conventional fluorescent optical probes and can provide additional insight into complex biological systems.

### Acknowledgments

B M R acknowledges support from the National Institutes of Health through Grants R01CA138509 and R01GM142012.

## 12. Spectral histopathology: a diagnostic modality with an accuracy exceeding that of combined classical histopathology and immunohistochemistry

### Max Diem

Northeastern University and CIRECA LLC, United States of America

### Status

Spectral histopathology (SHP) [137, 138] is an optical, multispectral imaging technique utilizing the rich infrared fingerprint region (2.5–12 *µ*m wavelength) to identify differences in the biochemical composition of tissue voxels measuring approximately 10 × 10 × 5 *µ*m^3^. The amount of data collected from a 1 mm^2^ area of tissue at 1500 infrared (colour) channels exceeds 10^7^ discrete compositional data. This huge amount of data acquired from even a small piece of tissue allows multivariate analysis *via* self-learning algorithms to render pathological assessment with an accuracy surpassing that of classical pathology.

SHP is based on the detection of changes in biochemical composition [139], rather than morphological features, and is therefore more akin to methods such as MALDI-TOF (matrix assisted laser desorption/ ionization-time of flight mass spectrometry) imaging [140]. SHP demonstrated that changes in tissue morphological features observed in classical pathology are accompanied by changes in the biochemical composition at the cellular level [141]. Thus, these imaging methods provide novel insight into biochemical changes due to disease and—since SHP is based on a physical measurement—it renders diagnoses on a more objective and reproducible basis than methods based on assessing cell morphology and tissue architecture.

Several large studies [139, 141–144] of archived patient lung tissue, in collaboration between the Department of Thoracic Surgery of the City of Hope (COH) Cancer Center in Duarte, CA, the Department of Pathology at the University of Massachusetts Medical School (UMP), Worcester, MA, and CIRECA, LLC, (then in Cambridge, MA) demonstrated that SHP can be used for the distinction of small cell lung carcinomas (SCLC), adenocarcinomas (ADCs) and squamous cell carcinomas (SqCCs) of the lung with accumulated accuracies of better than 90%. In addition, it was found that SHP can be used to resolve interobserver differences in lung pathology [142] for tissue core sections for which the COH and UMP diagnoses differed (about 15% of all cases). In these instances, SHP results mostly agree with those of IHC, considered the gold standard for discriminating non-small cell lung cancers. This is since both SHP and IHC are sensitive to the presence of particular markers that are invisible in classical histopathology [145]. Furthermore, SHP reliably classified mixed tumours such as adeno-squamous carcinomas (AdSqCC). These mixed tumours often exhibit regions that show characteristic signs of either of the two cancer classes.

### Current and future challenges

Next, some representative results will be presented that show the potential and limitations of the SHP technology. These results were taken from comprehensive studies published elsewhere [142].

**Figure 18.**
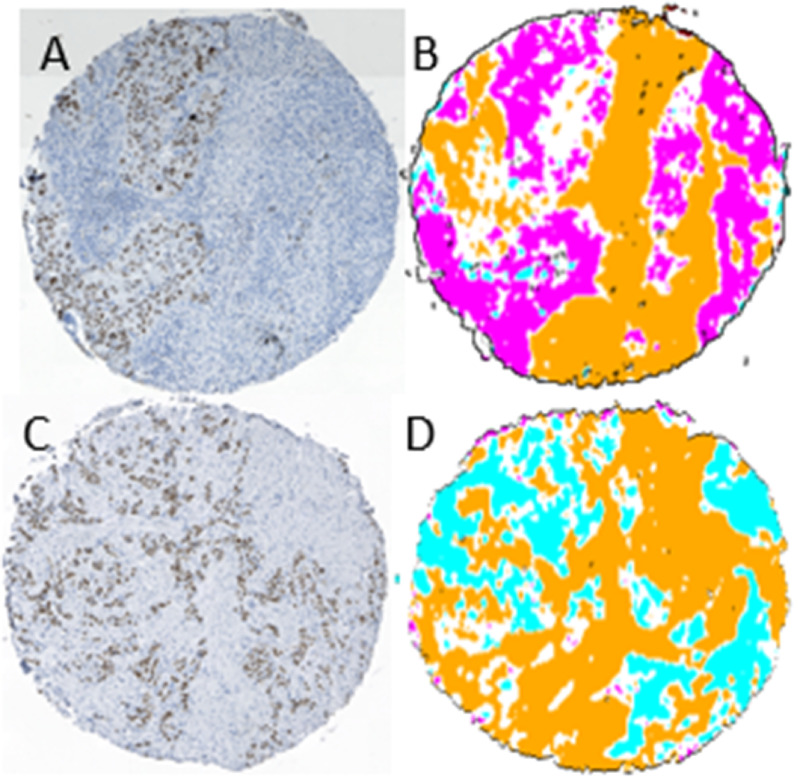
ADC vs. SqCC classification. (A) TTF-1-stained tissue core diagnosed by pathology as SqCC. (B) SHP prediction image (C) p40 stained tissue core diagnosed by pathology as ADC. (D) SHP prediction image. (Core diameter: ∼1.5 mm) Colour code for SHP prediction: orange: cancer adjacent normal; blue: SqCC; pink: ADC.

In the two tissue core sections shown in figure 18, the pathological diagnoses from COH and UMP disagreed in terms of the ADC vs. SqCC assignment. In both cases, SHP agreed with the IHC results: the SHP predictions (panels (B) and (D) of figure 18) agree with the IHC positive areas (panel (A): TTF-1 stain for ADC and panel (C): p40 stain for SqCC) not only in the gross diagnosis, but also in the regions that show positive IHC response. This observation re- emphasizes the statement above that SHP and IHC are sensitive to the presence of specific cancer markers.

**Figure 19.**
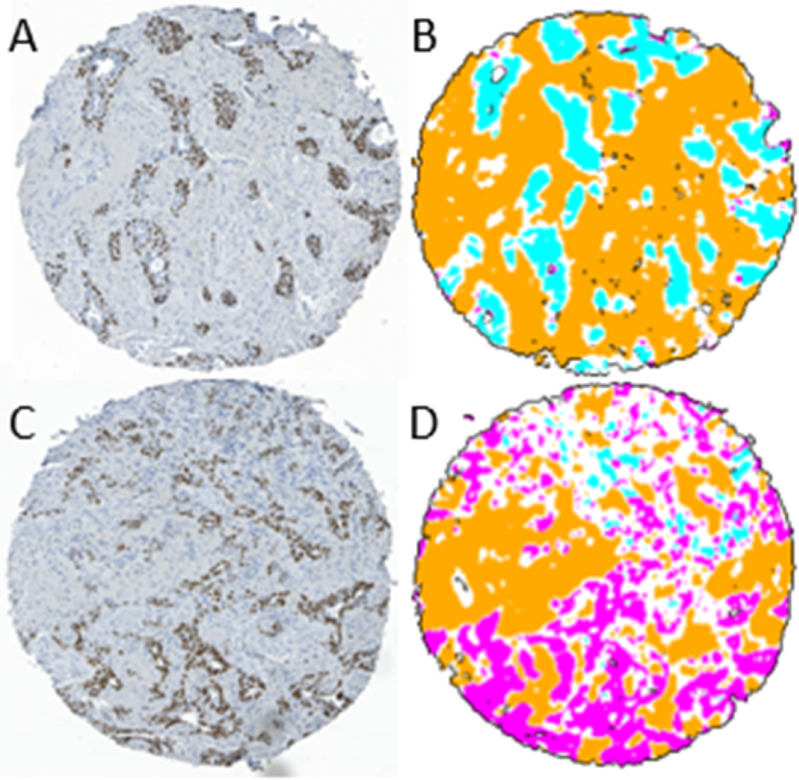
Adeno-squamous (AdSqCC) case study. (A) p40-stained tissue core from patient diagnosed with AdSqCC. (B) SHP prediction image. (C) TTF-1- stained tissue core from same patient, different location. (D) SHP prediction image. Colour codes as in figure 18. (Core diameter: ∼1.5 mm).

Figure 19 shows two tissue cores from the same patient biopsy, but from different regions of the tumour. Both sections were classified by pathology as AdSqCC. In both cores, IHC and SHP results indicate that the cores are nearly entirely SqCC (panels (A) and (B)) and ADC (panels (C) and (D)), dependent on the exact location from which the tissue core was collected within the tumour mass. This result indicates a ‘biphasic’ AdSqCC where cells from areas that are clearly ADC and cells that are clearly SqCC merge at the margins of separate tumours and create regions of mixed cancer. The other description (that is no longer recognized within the WHO classification system) used the term ‘admixed’ AdSqCC, in which anaplastic tumour cells that may arise from multipotent stem cells show no microscopic evidence of squamous or glandular differentiation [146].

The examples shown in figures 18 and 19 point to the advantages of the SHP methodology which is based on reproducible, machine-based data and analysis by self-learning multivariate algorithms, and is totally independent of cell morphology, staining patterns and tissue architecture. The overall accuracies in the classification of SCLC, ADC and SqCC (99.6%, 92.2 and 91.6%, respectively) increased during a five-year period these studies were carried out mostly due to increased understanding of the number of tissue classes required for reliable algorithm training. This involved the distinction between truly normal and cancer adjacent normal tissue classes, and the use of IHC to verify pathological diagnoses. These results pave the way toward a wider application of this technology once certain operational caveats discussed in the next section are addressed.

### Advances in science and technology to meet challenges

There are two major aspects that need to be addressed for this technology to become more accepted in medical practices. The first of these is a matter of instrumentation for the acquisition of spectral data. Present commercially available infrared micro-spectrometers are based on interferometric methodology with cryogenic semi-conductor array detectors, or quantum cascade laser-based (QCL) systems with micro-bolometer detector arrays. The former of these methods is generally adequate in terms of data quality and reproducibility, but is too slow by at least an order of magnitude for large scale medical applications. QCL-based micro-spectrometers offer many advantages over interferometric instruments, but are at present plagued by coherence-induced artifacts and high prize, that have prevented their wide applications [147]. Collaborations between academic and industrial research will be required to address these instrumental issues.

The second aspect of methodology improvement involves the training of self-learning multivariate algorithms for the analysis of the hyperspectral datasets. Different research groups have used support vector machines, deep learning neural networks (NNs), linear discriminant analysis algorithms or other methods for this analysis. While the choice of the mathematical method appears to be less important (all of them produce comparable accuracies), the training of these algorithms is a task that requires more attention. First, the number of patients in the training and test set must fulfil standards of general medical statistics [143], and many reported results have ignored this point. Second, the aim of some research projects ‘…to detect cancer by spectral methods…’ is too narrow, since the detection of cancer can be performed very well, indeed, by classical pathology, and spectral methods must be gauged by a much higher standard, and must include the cancer type, the tumour micro-environment, necrosis and detection of immune cell activation. Inclusion of such effects require a very close cooperation with pathology, using immune-histochemistry or other advanced methods in modern histology and oncology to correlate the spectral data. This includes the use of relating the spectral data to the presence of cancer markers and/or their surrogates [145].

### Concluding remarks

The results presented herein demonstrate that SHP delivers a level of diagnostic accuracy that matches that of classical histopathology combined with immunohistochemistry. This very high accuracy results from the use of inherent, spectral (optical) signatures which are manifestations of the biochemical composition of tissue pixels. These signatures can be observed with a very high degree of reproducibility.

The use of multivariate mathematical analysis transforms the observed raw spectral datasets into images that depict heterogeneity in a tumour, tumour types and sub-types, the effect of a cancerous lesion on its surroundings, and the presence of tumour-infiltrating immune cells. The spectral signatures from annotated regions of tissue have been used to train multivariate algorithms for the unsupervised diagnostics of tissue samples. Several research groups have reported such analyses for different tissue types and diseases [148–151].

## Data Availability

No new data were created or analysed in this study.
